# Combination of Transcriptomic, Proteomic, and Metabolomic Analysis Reveals the Ripening Mechanism of Banana Pulp

**DOI:** 10.3390/biom9100523

**Published:** 2019-09-23

**Authors:** Taotao Li, Ze Yun, Qixian Wu, Hongxia Qu, Xuewu Duan, Yueming Jiang

**Affiliations:** 1 Key Laboratory of Post-Harvest Handling of Fruits, Ministry of Agriculture/Guangdong Provincial Key Laboratory of Applied Botany/Key Laboratory of Plant Resources Conservation and Sustainable Utilization, South China Botanical Garden, Chinese Academy of Sciences, Guangzhou 510650, China; taotaoli@scbg.ac.cn (T.L.); yunze@scbg.ac.cn (Z.Y.); wuqixian@scbg.ac.cn (Q.W.); q-hxia@scbg.ac.cn (H.Q.); ymjiang@scbg.ac.cn (Y.J.); 2 University of Chinese Academy of Sciences, Beijing 100039, China

**Keywords:** fruit, softening, auxin, proteomic, transcriptomic, signal transduction

## Abstract

The banana is one of the most important fruits in the world. Bananas undergo a rapid ripening process after harvest, resulting in a short shelf. In this study, the mechanism underlying pulp ripening of harvested bananas was investigated using integrated transcriptomic, proteomic, and metabolomic analysis. Ribonucleic acid sequencing (RNA-Seq) revealed that a great number of genes related to transcriptional regulation, signal transduction, cell wall modification, and secondary metabolism were up-regulated during pulp ripening. At the protein level, 84 proteins were differentially expressed during pulp ripening, most of which were associated with energy metabolism, oxidation-reduction, cell wall metabolism, and starch degradation. According to partial least squares discriminant analysis, 33 proteins were identified as potential markers for separating different ripening stages of the fruit. In addition to ethylene’s central role, auxin signal transduction might be involved in regulating pulp ripening. Moreover, secondary metabolism, energy metabolism, and the protein metabolic process also played an important role in pulp ripening. In all, this study provided a better understanding of pulp ripening of harvested bananas.

## 1. Introduction

The banana (*Musa spp*.) is one of the most important fruits in the world [1,2]. The banana is a typical climacteric fruit and undergoes a rapid ripening process after harvest [3]. Pulp softening is the typical characteristic of banana ripening, which is essential for nutritional and sensorial quality of the fruit [4]. However, over-softening accelerates the senescence processes, which causes great economic losses. Therefore, a better understanding of the regulation of pulp ripening helps to develop strategies to reduce harvest loss.

Currently, studies on banana fruit ripening and softening have mainly focused on biochemical changes and transcriptional regulation [5,6,7,8]. Duan et al. reported that modifications in compositions and glycosyl linkages and molecular mass of pectin polysaccharide were responsible for banana fruit softening [9]. Trivedi and Nath proposed that non-enzymatic process of cell wall depolymerization mediated by expansins is involved in banana softening [10]. Transcriptional regulation plays a vital role in fruit ripening. Several transcription factors have been identified as ripening regulators of banana fruit. Xiao et al. showed that MaERF9 and MaERF11 act as activator and repressor to participate in transcriptional regulation of banana fruit ripening, respectively [11]. Kuang et al. reported that MaDREB2 is involved in regulating banana fruit ripening as both transcriptional activator and repressor [12]. In addition, other transcription factors, including MYB, NF-Y, BZR1/2, NAC, AP2/EREBP, WRKY, and C2H2-type zinc finge were also reported to be implicated in the regulation of banana fruit ripening. [13,14,15,16]. However, understanding of other mechanisms underlying the ripening and softening mechanism of banana fruit is still limited.

Recently, “omic” technologies, such as transcriptomics, proteomics, and metabolomics, were widely used in postharvest fruit to reveal the complexity of the development, ripening, and senescence mechanisms of different fruits [17]. As for the banana, fruit ripening was investigated by the comparative proteomics method [18]. However, due to a lack of genome information, only limited differential–expressed proteins in banana pulp were identified. D’Hont et al. [19] also performed transcriptome analysis of banana pulp and peel and found that acetylene treatment induces changes in gene expression independent of the ability to initiate ripening in response to ethylene. Asif et al. [16] then focused on the differences of gene expression between ripe and unripe banana pulp tissue and identified major metabolic networks involved in the fruit ripening process. Unfortunately, these transcriptome studies did not reflect the dynamic changes in gene expression of banana pulp. Additionally, transcriptomic or proteomic analysis alone is not sufficient to understand the complex mechanism underlying banana fruit ripening and quality deterioration. Our recent research highlighted a better understanding of the mechanism underlying banana peel ripening based on transcriptomic, proteomic, and metabolomics analysis [20]. Therefore, the combined omic technologies could provide us with a better knowledge of banana pulp ripening at the whole-genome level.

In this study, transcriptomic, proteomic, and metabolomic in combination with ultrastructure analysis were conducted for an integrated analysis of banana pulp at different ripening stages. Dynamic analysis of differentially expressed genes and proteins help us learn the specific expression pattern of different softening stage. In all, our results provided comprehensive knowledge of the complex mechanisms that regulate banana pulp ripening/softening.

## 2. Materials and Methods

### 2.1. Sample Collection

Bananas (*Musa spp*. cv. Brazil) were harvested around 110 days after flowering from a commercial orchard in Huizhou, Guangdong, China. Fruit with uniform shape, color, and size were selected, washed with fresh water, and then dipped for 3 min in 0.1% Sportak (prochloraz, Bayer) fungicide solution to control postharvest decay. The fruit were allowed to air-dry. The pulp of different ripening stages of fruit, i.e., 0 d (F1), 12 d (F2), 16 d (F3), and 18 d (F4), were sampled with liquid N_2_ and then stored at −80 °C for further analysis.

### 2.2. Banana Maturation Characters Determination

Fruit firmness was determined using a penetrometer (Model GY-3, Zhejiang Scientific Instruments, Zhejiang, China) and expressed as *N*. Respiration rate was measured according to our previous method and express as mg CO_2_ kg^−1^ h^−1^ [21]. 

### 2.3. Scanning Electron Microscopy and Transmission Electron Microscopy Analysis

Banana pulp of different stages was cut to get several segments (about 5 × 10 mm) for scanning electron microscopy (SEM) and transmission electron microscopy (TEM) analysis using our previous methods [22].

### 2.4. RNA Extraction and Digital Gene-Expression Profiling

Frozen pulp tissues (10 g) corresponding to four stages were used for RNA extraction using the method described by Kuang et al. [12]. DNA contamination was eliminated with DNaseI digestion using the RNase-free kit (Promega, Madison, WI, USA). 

RNA-Seq (Quantification) analysis was undertaken at four different stages of banana pulp softening: F1, F2, F3, and F4. RNA samples were sent to the Beijing Genomics Institute (Shenzhen, China) for analysis. The mRNA was enriched and used to construct the cDNA library. Then, the library products were sequenced via Illumina HiSeqTM 2000 (Illumina Inc, San Diego, CA, USA). The reads containing adaptor sequences, more than 10% unknown bases, or more than low quality base (base with quality value ≤5) were removed to obtain clean reads. After assessment of sequencing, the expression level for each gene was determined according to the method of Mortazavi et al. [23]. Fold change ≥2 with false discovery rate (FDR) ≤ 0.001 was set as the threshold for the differentially expressed genes. Figure S1 provides the details for the pipeline that showed all the analysis done in this study.

### 2.5. Quantitative Real-Time PCR Validation

Quantitative real-time polymerase chain reaction (qRT-PCR) was conducted according to our previous research [24]. The primer sequences were provided in Table S1.

### 2.6. Protein Extraction, 2-D Gel Electrophoresis, Gel Staining, and Image Analysis

Total proteins extraction, 2-D gel electrophoresis, gel staining, image analysis, and data handling was conducted according to our previous research [25]. 

### 2.7. In-Gel Tryptic Digestion, MS Analysis, and Database Searching

The differently accumulated protein spots were manually excised and then subjected to in-gel tryptic digestion and mass spectrometry (MS) analysis according to Li et al. [25]. The banana genome (http://banana-genome.cirad.fr/home) was used to obtain the identity of the proteins.

### 2.8. Correlation Analysis of Transcriptomics and Proteomics 

The correlation between proteomics and transcriptomics was analyzed for differentially expressed proteins and genes in the whole library. R version 3.3.2 [26] was also used for principal component analysis of differentially genes and proteins in transcriptomic and proteomic data.

### 2.9. Primary Metabolic Profiling

Samples collected at different periods of banana pulp ripening (F1, F2, F3 and F4) were used for the differential primary metabolic profiling analysis using the gas chromatography-mass spectrometry (GC-MS) system according to Yun et al. [27]. Three biological replicates were conducted for primary metabolic analysis.

### 2.10. Least Squares Discriminant Analysis

Protein accumulation was subjected to partial least squares discriminant analysis (PLS-DA) using SIMCA (version 15.0) according to our previous research [28]. The proteins differentially accumulated during ripening process toward the separation in PLS-DA model was further analyzed where proteins with variable importance (VIP) values exceeding 1.0 and *p* < 0.05 were selected as the cut-off.

### 2.11. Statistical Analysis

Data for each sample was statistically analyzed using SPSS (version 7.5, IBM Inc., Chicago, IL, USA) by one-way analysis of variance (ANOVA) with least significant differences at 0.05 level.

## 3. Results

### 3.1. Phenotype Change of Ripening Banana Fruit

**Figure 1:**
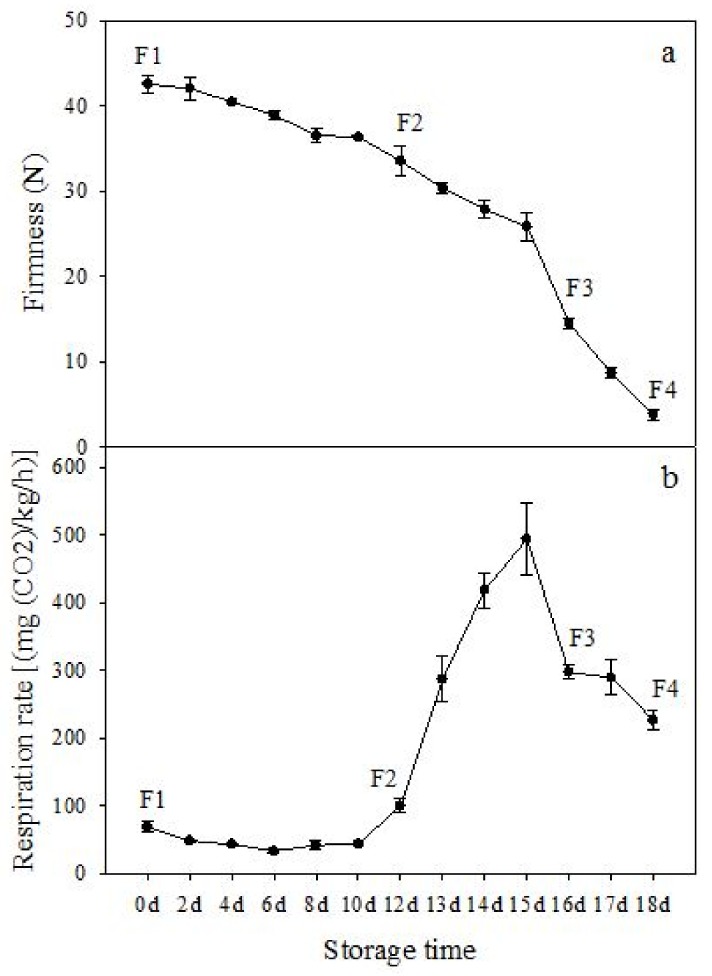
Changes in firmness (**a**) and respiration rate (**b**) of banana fruit at different ripening stage. Each value represents the mean ± standard error (SE) of three replicates. The least significant difference (LSD) (*p* = 0.05) was calculated for mean separation.

The firmness and respiration rate change that occurred in the ripening process of bananas can be observed in Figure 1. The firmness of banana pulp decreased slightly at stage F2. At stage F3, the firmness decreased sharply and subsequently developed to fully ripening stage F4 (Figure 1a). For respiration rate, at stage F2, the respiration rate began to rise and peaked at 15 days, after which the samples were collected at F3 (16 days) and fully ripening stage F4 (Figure 1b).

**Figure 2:**
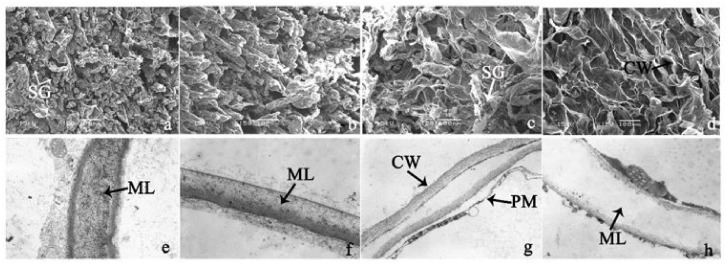
The observation on amyloplasts at different stages of banana flesh ripening. (**a–d**) scanning electron microscopy (SEM) image of F1, F2, F3, and F4, respectively; (**e**–**h**) transmission electron microscopy (TEM) image of F1, F2, F3, and F4, respectively. SG: starch grain; CW: cell wall; ML: middle lamella; PM: plasma membrane.

To investigate the cell structural changes of banana pulp during the ripening period, we conducted SEM and TEM analysis. As shown in Figure 2, the number of starch grain and the ultrastructure of pulp cell changed evidently along with fruit softening. SEM results showed that the number of starch grain gradually decreased due to the degradation of starch (Figure 2a–d). At stage F4, the starch grain almost disappeared, and the cell wall collapsed (Figure 2d). For the TEM image, at the early stage of pulp ripening, the cell wall structure and middle lamella (ML) remained intact (Figure 2e,f). Along with the ripening of banana pulp, the ML began to disrupt at stage F3, and the cell wall partly disassembled (Figure 2g). Finally, the ML almost disappeared, and the cell wall depolymerized entirely, at stage F4 (Figure 2h). Interestingly, the mitochondria remained unbroken during the whole ripening process (Figure S2).

### 3.2. Digital Gene-Expression Profiling of Specific Genes of Banana Fruit

To further investigate molecular change in the banana pulp at different stages, differentially expressed genes were analyzed using RNA-seq. Compared with F1, 14 genes were down-regulated and 352 were up-regulated at F2, 130 down-regulated and 483 up-regulated at F3, and 34 down-regulated and 269 up-regulated at F4. Additionally, qRT-PCR was used for validation of the RNA-Seq data. The qRT-PCR expression patterns and RNA-Seq data were similar for most of the 18 randomly selected genes (Figure S3), which further confirmed the reliability of the RNA-Seq data.

### 3.3. Two-DE Analysis of Banana Pulp

**Figure 3:**
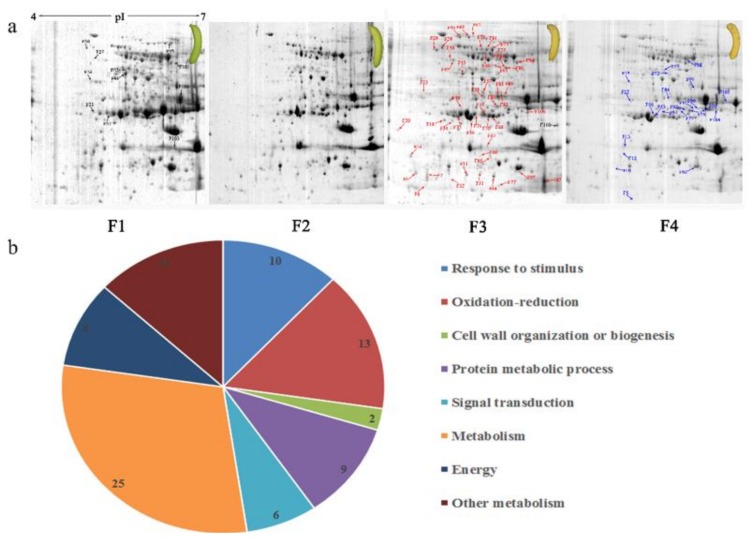
Proteomic analysis of banana pulp from different ripening stages of fruits. (**a**) Representative two-dimensional electrophoresis profiles and distribution of identified proteins. (**b**) Functional categorization of identified proteins using Blast2GO program. F1, F2, F3, and F4 represent different ripening stages of fruits, respectively. Red arrows and green arrows indicate the up-regulated and down-regulated proteins compared with F1, respectively. Black arrows indicate the proteins with complicated expression pattern. pI: Isoelectric point.

Protein spots were separated with isoelectric point (pI) 4–7 and representative gels were shown in Figure 3a. A two-fold threshold was used to judge the changes in abundance as significant for the regulated proteins during banana pulp ripening. Based on these criteria, a total of 94 differentially expressed protein spots were identified by matrix-assisted laser desorption/ionization time-of-flight (MALDI-TOF) MS/MS, and 84 spots were successfully identified on the basis of tryptic peptide sequences according to banana genome. Most of the identified proteins were up-regulated. In detail, compared with F1, 53, 58, and 57 proteins were up-regulated, and 31, 26, and 27 proteins were down-regulated in F2, F3, and F4, respectively. The specific locations of proteins on two-dimensional gel electrophoresis (2-DE) gels are shown in the gels in Figure 3a, and detailed information of each protein is given in Table 1. According to functional categorization of the 84 identified proteins, these differentially expressed proteins were mainly involved in metabolism, oxidation-reduction process, protein metabolic process, energy, and so on (Figure 3b).

### 3.4. Correlation Analysis of Proteomics and Transcriptomics

A correlation analysis was also performed between the differentially accumulated proteins and genes in the whole library. A total of 84 protein:mRNA ratios across all three stages were shown in Figure S3 and the correlation coefficient of different stage was determined respectively as F2: 0.195395, F3: 0.318828, and F4: 0.416578 (Figure S4), which indicated a positive directional correlation between the mRNA and protein abundance ratios, especially there was a significant correlation in F3 with *p*-value = 0.003307, F4 with *p*-value = 8.955 × 10^−5^, and marginal correlation in F2 with *p*-value = 0.07488. At the F2 stage, most spots fell mainly in quadrants b, e, and h, which showed that a few spots reflected significant changes at both the transcript and protein levels. Only three spots appeared in quadrant c, and six spots appeared in g. The F3 stage was characterized by relatively more spots falling into the quadrants a, c, g, and i compared with the F2 and F4 stages, where the protein and transcript levels were substantially different. However, contrasting levels were observed for many genes, which exhibited down-regulated expression but up-regulation of its protein abundance. At the F4 stage, the correlation coefficient was highest, and most spots were observed in the quadrants c and g, where more spots showed up/down-regulation at both levels (Figure S4). In all, our results showed a strong correlation between the transcripts and proteins. The spots that showed significant changes at only one level, the transcript or protein level (shown in quadrants d and f), demonstrated that there was a substantial degree of post-transcriptional translational and/or posttranslational regulatory activity during banana fruit ripening.

### 3.5. Partial Least Squares Discriminant Analysis of Differentially Accumulated Proteins

**Figure 4:**
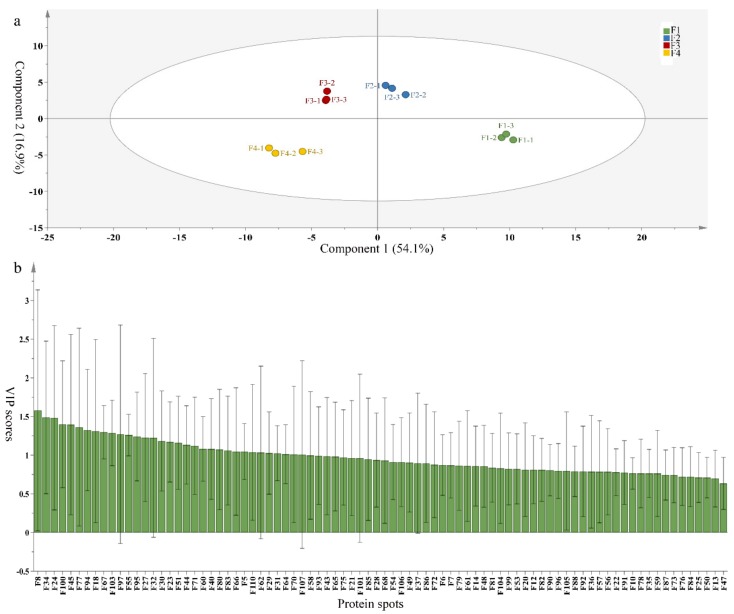
Partial least squares discriminant analysis (PLS-DA) analysis of differentially accumulated proteins. (**a**) PLS-DA score plot; (**b**) Identified proteins ranked by variable importance (VIP) scores that towards component 1 in PLS-DA analysis.

To compare differences in proteins from banana fruit at different ripening stages, PLS-DA analysis was carried out. The scores and correlation loadings obtained for the first two latent components of the calculated PLS-DA model were combined in a bi-plot. The first two components of PLS-DA accounted for 71% of the total variance among samples (Figure 4a). As shown in Figure 4a, fruits were separated completely along component 1 and component 2. Additionally, we also calculated the values of variable importance (VIP) scores for the identified proteins to identify proteins that could explain separation of banana fruit at different ripening stages. The VIP is used to explain the weight of the independent variable in explaining the dependent variable. As reported in our previous research [28], the variable with VIP values exceeding 1.0 are considered to have important roles in the PLS-DA discriminant process. In this study, 33 proteins with VIP exceeding 1 (F8, F34, F24, F100, F45, etc.) were identified, which could explain separation of banana fruit at different ripening stages (Figure 4b).

### 3.6. Differentially Accumulated Primary Metabolites in the Process of Banana pulp Ripening

We also investigated the changes in primary metabolic composition during banana pulp ripening. A total of 40 identified metabolites were monitored in the same sample sets using GC-MS, including mainly alcohols, sugars, organic acids, and fatty acids (Table 2), which showed differences in levels during fruit ripening. An analysis of significance with ANOVA (*p* < 0.05) indicated that 19 detected metabolites, falling into mostly sugars and alcohols, were found to be significantly regulated in different stage of banana pulp maturation when compared to F1 (0 d). In addition, the profiling of primary metabolism indicated that almost all soluble sugars exhibited significance increases during fruit maturation, especially for galactopyranose, which increased more than 680-fold at stage F4. At the same time, the d-(-)-Fructopyranose was greatly up-regulated with more than 170 folds at stage F4. The accumulations of α-d-Mannopyranoside and d-(+)-Turanose also increased during the pulp ripening process (Table 2).

### 3.7. Differentially Expressed Signal Transduction and Transcriptional Regulation Related Genes and Proteins During Banana Pulp Softening

**Figure 5:**
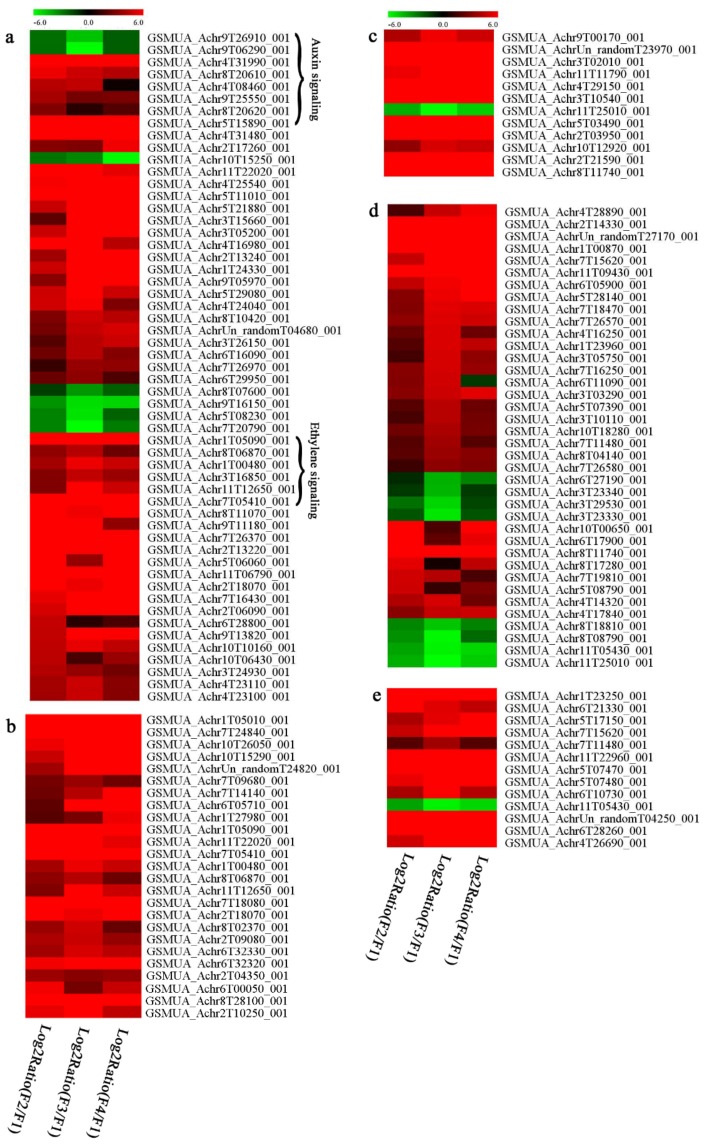
Differentially expressed genes of signal transduction related (**a**), transcriptional regulators (**b**), secondary metabolism (**c)**, sugar metabolism (**d**), and cell wall related (**e**) in the process of banana flesh ripening. Differentially expressed genes were analyzed using comparative transcriptomic technology and the logarithm of the ratio between two samples with 2 base was present in this figure. Green color shows down-regulated genes, and red color shows up-regulated genes.

In signal transduction, among the differentially expressed genes, there were 22 genes up-regulated without any down-regulation at F2; for F3, 30 genes were up-regulated and six genes down-regulated; and at stage F4, 16 genes were up-regulated with only two genes down-regulated (Figure 5a). All these genes were clustered using BiNGO 2.44 (Figure S5). Most of these genes were clustered into the auxin signaling pathway, such as GSMUA_Achr4T08460_001 (*indole-3-acetic acid-amido synthetase*), GSMUA_Achr5T15890_001 (*auxin efflux carrier component*), GSMUA_Achr4T31990_001 (*Auxin-responsive family protein*), and GSMUA_Achr8T20620_001 (*Auxin-induced protein 22D*). Among genes encoding the auxin responsive protein, there were three up-regulated and two down-regulated. In all, the auxin signal transduction–related genes were rather active during the ripening process, and there was a crosstalk between auxin and ethylene signaling pathway (Figure S5). Most of the differentially expressed genes of ethylene signaling pathway were greatly induced at F3 (Figure 5a) such as ethylene-responsive transcription factor 4, ethylene-responsive transcription factor ERF105, ethylene-responsive protein related, and ethylene-responsive transcription factor ERF021. It seemed that the auxin signal appeared earlier than the ethylene signaling. For protein level, auxin-induced protein PCNT115 (spot F93) was down-regulated, and IAA-amino acid hydrolase ILR1-like 1 (spot F87) was up-regulated (Table 1).

Four genes of ethylene-responsive transcription factor (ERF) were only observed differentially expressed at the stage of F3 and showed great up-regulation; only one gene of *ERF* (GSMUA_Achr1T05090_001) was up-regulated in F2, and GSMUA_Achr7T05410_001 in F4; no down-regulation of ERF was detected during the whole stage of banana ripening (Figure 5b).

Two genes of *MYB* (GSMUA_Achr7T18080_001 and GSMUA_Achr2T18070_001) and two genes encoding MADS-box transcription factor (GSMUA_Achr8T28100_001 and GSMUA_Achr2T10250_001) were up-regulated at all stages of banana ripening, and no down-regulation was detected. Additionally, another gene encoding MADS-box transcription factor (GSMUA_Achr2T04350_001) was differently expressed with upregulation in F2 and F3; GSMUA_Achr6T00050_001 was up-regulated only at F2 stage.

For the WRKY TF family, more genes were up-regulated at the stage F4 with seven genes; there were five genes and only one gene up-regulated in F3 and F2, respectively (Figure 5b).

According to Figure 5b, one differentially expressed gene of *NAC* (GSMUA_Achr6T32320_001) was up-regulated during the whole stage of banana pulp ripening, and another two genes (GSMUA_Achr2T09080_001 and GSMUA_Achr6T32330_001) were up-regulated in the early stage of banana pulp ripening. Our results suggested that more TFs were up-regulated in stage F3, and these TFs might exit an important role in banana pulp softening.

### 3.8. Metabolism Related Genes and Proteins were Differentially Expressed During Banana Pulp Softening

In secondary metabolism, 136 genes and proteins were differentially expressed in banana senescence. In F2, 63 genes were up-regulated and two down-regulated; 68 genes were up-regulated and nine genes were down-regulated in F3; for F4, 47 genes were up-regulated with three downregulations (Table S2). Interestingly, only 11 genes were up-regulated and only one gene of *glycosyl transferase* (GSMUA_Achr11T25010_001) was down-regulated in all stages (Figure 5c). The changes of genes expression suggested more metabolic changes happened in stage F3. 

In sugar metabolism, one gene of the β-amylase 3 and one gene of aldehyde dehydrogenase were up-regulated, while only two genes were down-regulated—*pectinesterase 3* and *glycosyl transferase*. Additionally, most genes were differentially expressed in stage F3 (Figure 5d), and these genes were mainly clustered into cell wall and starch metabolism using BinGo2.44 (Figure S6). For example, in the late stage of banana pulp ripening, the genes encoding soluble starch synthase (GSMUA_Achr3T03290_001), polygalacturonase (GSMUA_Achr9T16670_001), and pectinesterase/pectinesterase inhibitor (GSMUA_Achr7T15620_001, GSMUA_Achr3T05750_001) were greatly induced, which might accelerate the degrading of starch and cell wall polysaccharide. In contrast, the genes of *glucosidase* (GSMUA_Achr3T29530_001) and *glycosyl transferase* (GSMUA_Achr11T25010_001) were down-regulated in the late stage of ripening (Figure 5d). In addition, F3 was characterized as the most active stage in which the genes encoding starch and sucrose was differently expressed (Table S2).

The protein involved with starch metabolism, granule-bound starch synthase1, was up-regulated at the protein level. Strangely, starch phosphorylase, another important enzyme involved in starch degradation, was not identified in either the DGE or the proteomic data.

There were 13 proteins which were related to malate metabolism with only one protein (s-adenosylmethionine synthase 1) down-regulated. Fortunately, 3-isopropylmalate dehydrogenase was also identified in the proteomics data, and this protein was up-regulated during banana pulp ripening (Table 1). All these results indicated organic acid metabolism play an essential role during banana pulp maturation.

For cell wall metabolism, as shown in Figure 5e, 13 genes were differently expressed and four genes were up-regulated during the whole ripening process, including *expansin-A8* (GSMUA_Achr11T22960_001), *expansin-A2* (GSMUA_Achr5T07470_001), *pectate lyase15* (GSMUA_AchrUn_randomT04250_001), and *pectate lyase22* (GSMUA_Achr6T28260_001). Only one gene of *pectinesterase3* (GSMUA_Achr11T05430_001) was down-regulated in the banana ripening process. The component changes of the cell wall during the pulp ripening are indicated in Figure S7. Interestingly, in the signal transduction related genes, we also found that *XET* genes were greatly induced, including two genes (GSMUA_Achr5T21880_001, GSMUA_Achr3T05200_001) in stage F3 and F4 and one gene (GSMUA_Achr9T11180_001) in stage F2 and F3. GSMUA_Achr9T13820_001 was greatly up-regulated during all stages (Figure 5a). This suggests that there might be a crosstalk between signal transduction and cell wall metabolism.

There were also seven aquaporin genes differently expressed. Most of the genes were up-regulated, especially in stage F2, with four genes (GSMUA_Achr11T00590_001, GSMUA_Achr4T20780_001, GSMUA_Achr11T02240_001, and GSMUA_AchrUn_randomT06140_001). In stage F3, three genes (GSMUA_Achr9T28940_001, GSMUA_Achr8T12920_001, and GSMUA_Achr6T05830_001) were down-regulated, and one (GSMUA_Achr4T20780_001) was up-regulated. Only one gene (GSMUA_AchrUn_randomT06140_001) was differently expressed with upregulation in stage F4 (Table S2). For protein level, UTP--glucose-1-phosphate uridylyltransferase (spot F73) was identified with up-regulation (Table 1).

It is worth noting that two genes of Cytochrome P450 and one gene of *Trans-cinnamate 4-monooxygenase* (GSMUA_Achr10T12920_001) were greatly up-regulated in all stages of banana ripening, and two genes of *phenylalanine ammonia-lyase* (GSMUA_Achr5T03950_001 and GSMUA_Achr5T18560_001) were greatly up-regulated in stage F3. However, the gene of shikimate kinase (GSMUA_Achr9T27540_001), which was involved in phenylalanine metabolism, was down-regulated in stage F3. In all, the genes related to phenylpropanoid and lignin metabolism were very active during banana pulp ripening (Figure S8).

Additionally, a larger number of proteins predicted acidic endochitinase-like (spots F76, 82, 83, 94, and 97) *S*-adenosylmethionine synthase 5 (spot F86) and *S*-adenosylmethionine synthase1 (spot F88) were up-regulated at all stages (Table 1). These results showed that phenylalanine metabolism and acidic endochitinase-like played a vital role in banana pulp maturation.

**Figure 6:**
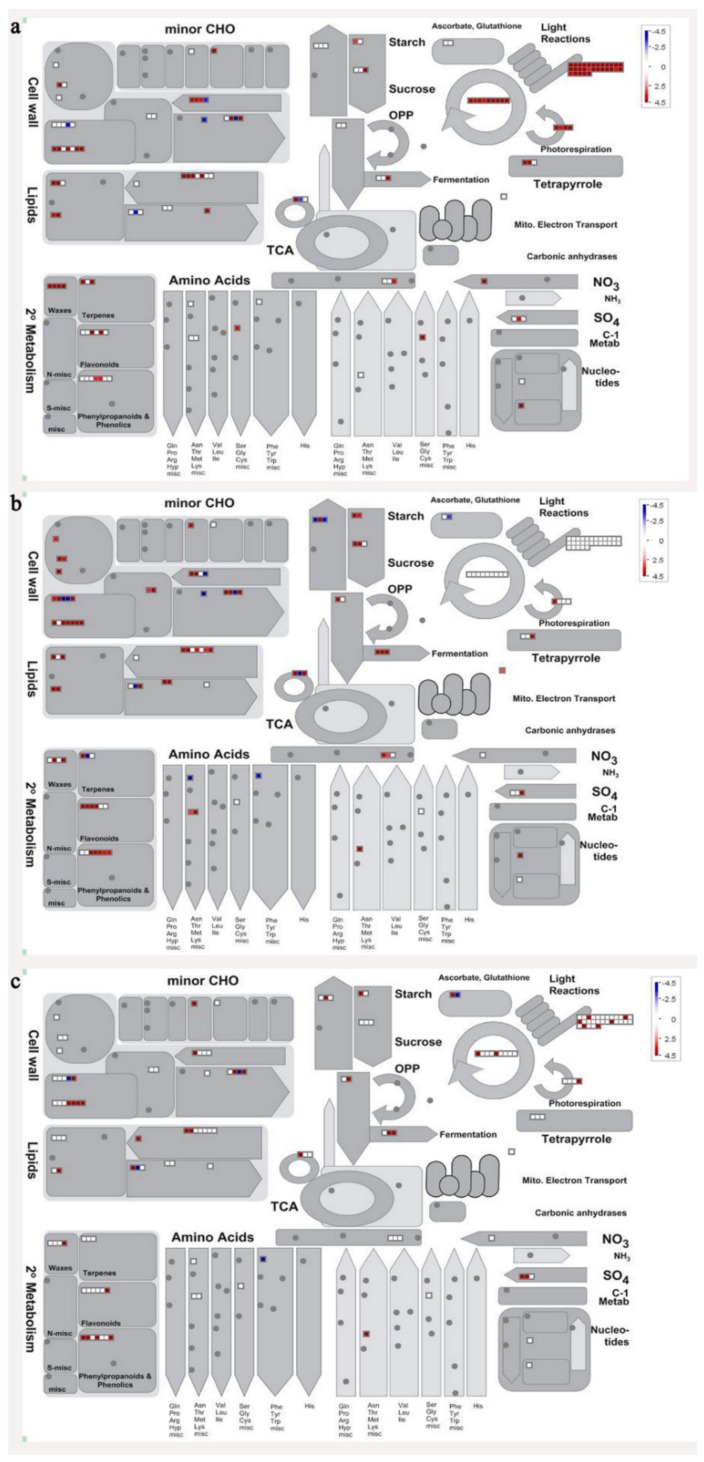
Schematic of the whole metabolism pathway using the MapMan visualization platform. The red or blue squares indicate the up- or down-regulated genes involved in the whole metabolic pathways. (**a**) F2/F1; (**b**) F3/F1; (**c**) F4/F1.

In order to visualize clearly the metabolic pathways that the differentially expressed genes were involved in, these genes were imported into the MapMan visualization platform (3.6.0RC1). As shown in Figure 6, the metabolic processes including cell wall metabolism, secondary metabolism, sugar metabolism, etc. were differentially regulated during banana pulp ripening process.

### 3.9. Oxidation-Reduction Process and Protein Metabolism Related Genes and Proteins were Differentially Expressed During Banana Pulp Softening

For oxidation-reduction process, four genes and 16 proteins were differentially expressed in the whole process of banana pulp ripening. Four genes, GSMUA_Achr7T24700_001 (*hydroxylase C887.15c*), GSMUA_Achr3T11750_001 (*3-oxoacyl-[acyl-carrier-protein] reductase*), GSMUA_Achr8T11740_001 (*Aldehyde dehydrogenase family 3 member F1*), and GSMUA_Achr10T12920_001 (*Trans-cinnamate 4-monooxygenase*) were up-regulated in the banana maturation process. Here, the up-regulation of aldehyde dehydrogenase gene suggested anaerobic respiration might play a vital role during banana pulp ripening.

Peroxidase was also observed to be differently expressed in certain stages, such as GSMUA_Achr5T29600_001 (*peroxidase 4*) and GSMUA_Achr3T16520_001 (*peroxidase 43*) up-regulated in stage F4, GSMUA_Achr5T25040_001 (*peroxidase 55*) and GSMUA_Achr6T21530_001 (peroxidase 52) up-regulated in stage F3, and GSMUA_Achr7T11330_001 (*peroxidase 15*) and GSMUA_Achr9T29540_001 (*peroxidase 12*) up-regulated in stage F2. Additionally, GSMUA_Achr5T20330_001 (*peroxidase5*) and GSMUA_Achr6T14620_001 (*peroxidase 52*) were both up-regulated in F2 and F3. On the other hand, GSMUA_Achr6T33120_001 (*L-ascorbate peroxidase*) was down-regulated in stages F3 and F4. Four proteins of L-ascorbate peroxidase (spots F37, F53, F56, and F68) were identified with different expression patterns (Table 1).

Protein disulfide oxidoreduction comprised a significant part in the oxidation-reduction process, especially with the ROS production during the fruit maturation. For protein level, protein disulfide-isomerase (spot F29) and one glutaredoxin protein (spot F70) were up-regulated in the proteomics data. Unfortunately, only one gene of *glutaredoxin* (GSMUA_Achr10T15090_001) was observed up-regulated in stage F2. Interestingly, three heat shock proteins (heat shock cognate 70 kDa protein) were up-regulated (Table 1).

## 4. Discussion

Despite the previous application of proteomics in banana ripening research [18], only a few spots were previously identified, and limited information was previously provided on the ripening mechanism of banana pulp. Also, previous transcriptomic research only focused on unripe and ripe banana fruit [16]. This study was undertaken to provide a dynamic map of the proteins and genes related to banana ripening using DEGs and proteomics. Additionally, the ultrastructure of banana pulp cells was investigated during the banana pulp softening period to comprehensively learn the ripening mechanisms of banana pulp. In the present study, expression of many genes and their corresponding proteins showed inconsistency, suggesting that posttranscriptional modification possibly plays a role in regulating banana fruit ripening.

A comparison between differentially expressed genes and proteins showed that the genes involved in signaling pathways, especially the auxin signaling pathway, were induced in stage F2, and the expression of most genes and proteins involved in ripening metabolic pathways was triggered in stages F3 and F4. It is suggested that banana pulp ripening is the result of a series of precise and coordinated physiological and biochemical processes, and the regulation of certain metabolism process occurred in certain stage. In addition, it is obvious that the F3 stage was characterized as having the most differentially regulated genes and proteins from transcriptomic and proteomic data. The respiration rate peak appeared before F3 at day 15 (Figure 1), so it is possible that the increase of respiration rate induced the sharp change of banana pulp metabolism. Some of the regulated genes in the whole metabolic pathways are shown in Figure 6 using MapMan. Among these, sugar metabolism and cell-wall biosynthesis might play a central role in banana pulp ripening. We also observed primary metabolism, which was significantly regulated during banana fruit ripening. All these data depicted a global picture of the metabolic network involved in banana fruit ripening.

### 4.1. Signal Transduction and Regulation During Banana Pulp Ripening

Ethylene was well proved to play a crucial role in banana fruit ripening and softening [11,12]. Reasonably, in the present study, one *ERF1B* gene was identified with upregulation as being involved in the ethylene signal transduction pathway. Interestingly, the protein of *S*-adenosylmethionine synthetase, which was involved in ethylene synthesis [11], was also observed with great up-regulation. Additionally, in the present study, several *serine/threonine-protein kinases* were greatly up-regulated in late stage of banana pulp ripening, including one *senescence-induced receptor-like serine/threonine-protein kinase* (GSMUA_Achr9T05970_001) (Figure 5a). Choudhury et al. reported that a Ser/Thr protein kinase is involved in the phosphorylation of 1-aminocyclopropane-1-carboxylic acid synthase 1 during banana fruit ripening [29]. It seemed that protein kinase mediated protein post-translational was involved in the acceleration of banana pulp ripening. It is worth noting that some of the MADS transcription factors, which might be involved in ethylene production and fruit ripening [30], were up-regulated during the pulp ripening in our present study. Interestingly, different members of *MADS* showed different expression patterns, such as GSMUA_Achr6T00050_001, which was only up-regulated in stage F2, and GSMUA_Achr2T10250_001, which was up-regulated during the whole ripening stage (Figure 5b). All these results indicated that some MADS might act in upstream of banana pulp ripening and others downstream of the ethylene signaling. Elitzur et al. [31] also observed that the expression of different *MaMADS* genes increased at different ripening stage. Our study suggested the possible role of MADS in banana pulp ripening, which was consistent with previous research [5]. Furthermore, the crosstalk between ethylene and other hormones including indole-3-acetic acid also influences fruit ripening [4]. Recently, the important roles of auxin in plant growth and development were well reviewed by Hagen [32]. Specifically, auxin also proved to play an important role in regulating fruit ripening [13,33]. Auxin response factors (ARFs) are repressors of auxin signaling, encodes a downstream component of auxin signaling [13]. In this study, three genes of *auxin-responsive family protein* (GSMUA_Achr4T31990_001, GSMUA_Achr8T20610_001, and GSMUA_Achr9T25550_001) were greatly up-regulated in the early stage of pulp maturation, while two were down-regulated in the late stage. Previous research indicated auxin response factors played important role in regulating auxin-responsive transcription in plants [34]. A recent study also showed that ARF works as positive regulators of tomato fruit ripening [13]. Moreover, other genes/proteins involved auxin synthesis and transport were also identified with up-regulation (Figure 5a, Table 1). Further, our proteomic results also showed that IAA-amino acid hydrolase ILR1-like 1 (F87) was largely up-regulated during the banana fruit ripening process (Table 1), which might result in an increase of the free IAA level. Furthermore, many genes of auxin signaling transduction were identified to be differentially expressed in different ripening stages (Figure 5a and Figure S5). In summary, the present data indicated that the auxin signal might also be involved in banana pulp ripening. Additionally, the application of IAA treatment could accelerate the ripening process of banana fruit [20].

Taken together, banana pulp ripening is likely to be regulated by the auxin and ERF-mediated ethylene signal pathways. Sugar signal might also play important role in regulating banana pulp ripening. Therefore, the stimulation of genes and proteins involved in signal transduction and regulation at early ripening stage might lead to the downstream process of banana pulp ripening (Figure S5).

### 4.2. Pulp Softening Played a Vital Role During Banana Ripening

Softening is an important determinant of banana ripening (Figure 1), which is related to cell wall disassembly. Cell wall disassembly during fruit ripening is the result of the dismantling of multiple polysaccharide networks by a diverse group of wall-modifying proteins, such as polygalacturonase (PG), pectin methylesterase (PME), and pectate lyase (PL) [35]. Cell wall–hydrolyzing enzymes are considered to be more active during this period.

Extensive structural modifications of pectin polymers caused by various cell wall modifying enzymes can result in the cohesion loosening between the cells. Then, cell wall polymers are less bound together and become highly hydrated [36]. In the present study, many genes involved in polysaccharide metabolism were differently expressed, especially in stage F3 (Table S2), which might induce the cell wall modification. For example, *polygalacturonase* genes, genes encoding pectinesterase/pectinesterase inhibitor, and two genes of *pectate lyase* were greatly up-regulated, which induced the degradation of pectin during banana ripening period (Figure 5d,e). In contrast, alpha-1, 4-galacturonosyltransferase (GSMUA_Achr11T25010_001: GAUT), which is the core of synthesis of homogalacturonan, was down-regulated (Figure 5d) and inhibited the synthesis of pectic polysaccharide. Meanwhile, the component changes of the cell wall indicated the increase of galactose and changes in glycosyl linkage (Table 2 and Figure S7). These changes were related to the depolymerization of pectin polysaccharides. UTP–glucose-1-phosphate uridylyltransferase (spot F73), and 3-isopropylmalate dehydrogenase (spot F47) was also identified in proteomic data (Table 1). We also found that the genes encoding cellulose metabolism were differently expressed in stage F3 (Table S2). Duan et al. [9] also reported that modifications in polysaccharide compositions and glycosyl linkages were responsible for banana fruit softening.

Xyloglucan endotransglycosylase/hydrolase (XTH) is another cell wall–modifying enzyme that can either cut and rejoin xyloglucan by xyloglucan endotransglucosylase action or hydrolyse xyloglucan by xyloglucan hydrolase action [37]. Therefore, XTHs contribute a lot to cell expansion. Importantly, XTHs were reported to be involved in the auxin and ethylene-mediated signaling and postharvest softening of many fruits [38,39]. Our results showed that *XET* genes were greatly induced in the late stage of banana pulp ripening (Figure 5a,e). According to the TEM image (Figure 2), ML almost disappeared at stage F4, which might suggest that the cell wall degradation accompanied with the pectin decomposition during the ripening process.

A previous study suggested that the tomato *expansin* gene family, LeExp2, is regulated by auxin [40]. Furthermore, expansins have been shown to play important roles in cell wall metabolism during the fruit ripening process by disruption of intermolecular adhesion [40]. In this study, two genes of *expansin-A8* and *expansin-A2* were up-regulated at all stages (Figure 5e). The change of these genes might be related to the great cell wall loosening of banana pulp (Figure 2). The results of this study suggested that auxin-mediated signal might control the cell wall degradation of banana flesh through the regulation of expansin and *XET* genes expression.

Lignins are a group of highly branched phenylpropanoid polymers in terrestrial plants. The presence of lignins limit the enzymatic degradation of cell walls and delignification could lead to the cell walls digestion [41]. MYB transcription factors have been widely reported as regulating lignin biosynthesis [42]. In addition, phenylalanine is the starting point of lignin biosynthesis, and *MYB* genes were reported to regulate the expression of the general phenylpropanoid biosynthetic genes and influence lignin biosynthesis [43]. In this study, a great number of critical genes in the phenylpropanoid and lignin metabolic pathway, fallen into *cytochrome P450*, s-adenosylmethionine synthase, *phenylalanine ammonia-lyase*, and *shikimate kinase* together with two genes of *MYB*, were up-regulated (Figure 6). *S*-adenosylmethionine synthase was also up-regulated at the protein level (Table 1). Our results suggested that the acceleration of the phenylpropanoid metabolic pathway could influence the metabolism of lignin during banana fruit ripening. Meanwhile, phenylpropanoid and lignin metabolism-related genes were both up-regulated at the same time points (Figure S8). Trans-cinnamate 4-monooxygenase, which is a key enzyme during lignin biosynthesis, was greatly up-regulated in all stages of banana ripening. The results indicated that lignin metabolism might also contribute to the cell wall alteration during banana fruit ripening.

Besides cell wall degradation, previous evidence supported the idea that fruit softening was also associated with the decline of fruit turgor [44]. For example, the aquaporin in strawberry (FaPIP1) is up-regulated, which coincides with fruit ripening [45]. Alleva et al. [46] also pointed out that cell wall degradation and water exchange mediated by aquaporins could be juxtaposed and even integrated events during strawberry ripening. As for the banana, aquaporin has been reported to be involved in environmental stresses [47], but no aquaporin was previously reported to be involved in banana ripening. Although the growth may stop during the banana ripening process, once ripening has started, the fruit continues accumulating water, which contributes to the maintenance of fruit turgor. The increment in aquaporin expression at an early stage might lead to fast water accumulation for the ripening fruit. In this study, stage F2 was characterized by the most *aquaporin* genes up-regulated, while stage F3 had more genes down-regulated. In the last stage, the banana flesh appeared fully ripened with only one *aquaporin* genes up-regulated (Table S2). However, our present data was not consistent with the results in the strawberry, in which the aquaporin genes expression pattern coincided with the ripening process [45]. Whether or not the cell wall disassembly or loss of cell turgor mediated by aquaporin contributes more to banana flesh softening is worth further research.

Starch-to-sugar metabolism contributes to pulp softening as well as sweetness development during banana ripening [4,48]. This suggests that β-amylase and granule-bound starch synthase (GBSS) in banana fruit could contribute to the maintenance of starch quality. In this study, the starch grain obviously decreased in the late stage of banana flesh ripening (Figure 2). Starch metabolism–related genes were also involved in the banana ripening process (Figure S6). Granule-bound starch synthase1 was significantly up-regulated in proteomic data, and PLS-DA also confirmed their important roles in separating fruit at different stages (Table 1, Figure 4). However, MapMan results showed that starch tended to be degraded into sucrose due to high expression level of starch degradation related genes, especially at F3 stage (Figure 6). Additionally, a recent review pointed out that hormonal and genetic regulation of starch degradation–related genes were involved in banana fruit ripening [4]. As described above, we also found that ethylene, auxin, and transcription factors were involved in banana ripening. Hence, our results further confirmed the effects of hormones and TF on the starch-to-sugar metabolism during banana softening. The SEM results also confirmed the starch degradation in pulp during the banana fruit ripening process (Figure 2). The evidence indicated that starch phosphorylase (EC 2.4.1.1) played an important role in starch degradation [18]. However, in the present study, no starch phosphorylase was identified among the differentially accumulated proteins. Zhu et al. [49] also showed that starch degradation in fruit was from the combined functions of the genes related to starch synthesis and degradation. In all, our results suggested that starch degradation contributed largely to the banana softening, and this process was an implicated reaction involving different enzymes. Meanwhile, the soluble sugars exhibited a high degree of variance during fruit ripening (Table 2), which was consistent with the results in strawberry [50]. Furthermore, morin treatment delayed the banana fruit ripening due to the delayed accumulation of soluble sugars (fructose, glucose, etc.) [51]. Altogether, the banana pulp softening process was characterized by great increase accumulation of soluble sugars (Table 2), which might be also attributed to the depolymerization of polysaccharide.

### 4.3. Energy Metabolism and Protein Metabolism were Involved in Banana Pulp Ripening

Glycolysis, the tricarboxylic acid cycle and oxidative phosphorylation are important energy-generating pathways and intracellular energy depletion or an ATP deficiency induces senescence of horticultural crops [21]. In this study, two *fructose-1,6-bisphosphatase* genes were significantly up-regulated at early ripening stage (Table S2). One enolase (spot F60) protein was also up-regulated during the ripening process (Table 1). In contrast, phosphoglycerate kinase (spot F59), which was responsible for ATP-generating in the glycolytic pathway, was significantly down-regulated (Table 1). In addition, PLS-DA analysis suggested that enolase (F60) might contribute more to fruit ripening than phosphoglycerate kinase (F59) (Figure 4b). These results suggested that glycolysis might tend to result in carbon conversion but not energy production at the early banana ripening stage. Previous research also documented that glycolytic and ethanol fermentation–related enzymes increased during fruit ripening at the transcript and protein levels [52]. In addition, D’Ambrosio et al. [52] suggested that the enhanced glycolytic pathway might provide carbon skeletons for the respiratory climax in ripe apricots. Based on these results, we postulated that upregulation of glycolysis-related genes at the early ripening stage possibly contributes to the carbon skeletons conservation for further consumption at the late stage of pulp ripening.

Meanwhile, our results also showed that proteins of ATP synthase subunit alpha and ATP synthase subunit beta (F62) were up-regulated (Table 1), but only one gene of *ATP synthase* (GSMUA_Achr9T13040_001) was up-regulated in stage F2 at the transcriptome level. PLS-DA analysis also confirmed the important role of F62 in banana fruit ripening (Figure 4). Moreover, TEM results showed that the ultrasturcture of the mitochondrion did not change and remained fully unbroken during the whole process of banana pulp ripening (Figure S2). This might suggest that maintaining mitochondria functionality is vital during banana flesh ripening due to its role in keeping the ATP status and carbon skeleton supply.

Excessive accumulation of ROS can result in oxidative damage to macromolecules that, in turn, causes a loss of structure and function or even cell death [53]. Wang et al. [54] reported that ubiquitin–proteasome systems were evidently induced during tomato fruit ripening. Given the upregulation of proteasome (spots F106 and F107), cysteine proteinase 2 (spot F35) in our proteomic data (Table 1), it was suggested that protein metabolic process was very active, which might induce the degradation of many functional proteins and subsequently disturb the ripening process. Importantly, F107 played an important role in separating fruit at different ripening stages according to PLS-DA analysis (Figure 4b). Therefore, protecting the proteins from degrading is important during the ripening process. Glutaredoxins are a multi-gene family of proteins that play a vital role in regulating redox balance through thiol-disulphide exchange reactions [55]. In the present study, one glutaredoxin (F70) and one protein disulfide-isomerase (F29) were identified and up-regulated in proteomics data (Table 1). Our results suggested that the oxidation of protein thiol residues might occur during the banana fruit ripening process. Further, Dergousova et al. [56] suggested that reducing system glutaredoxin/glutathione reductase could regulate the redox modifications of cysteine residues of Na,K-ATPase. The increased accumulation of glutaredoxin and protein disulfide-isomerase was possibly beneficial for reducing the sulfoxide of oxidized proteins. Additionally, HSPs were reported to play important roles in fruit development and ripening [57]. In this study, three heat shock proteins exhibited up-regulation during banana pulp ripening (Table 1), and eight heat shock protein transcripts were differentially accumulated, including one down-regulation in F2, three up-regulations and two down-regulations in F3, and two up-regulations in F4 (Table S2). PLS-DA analysis also confirmed their roles in the banana flesh ripening process (Figure 4b). In all, the protein metabolic process played important role in banana flesh ripening process, and we hypothesized that glutaredoxin and HSPs were vital to keep the enzymes or proteins from damage via post-translational modification during banana pulp ripening.

In short, banana pulp ripening was accompanied by ROS stress, which caused protein oxidation, especially for proteins containing thiol. Up-regulation of peroxidases and glutaredoxins help maintain protein function by protecting proteins from oxidative stress to indirectly regulate the pulp ripening. Post-translational modification via HSPs also played an important role in protecting proteins from degradation.

## 5. Conclusions

The proteomic and transcriptomic analysis accompanied with metabolic and ultrastructure analysis of banana pulp were reported. The analysis of the gene/protein expression changes indicated an important role for proteins and genes involved in cell wall metabolism, sugar metabolism, ROS, and energy metabolism in banana pulp softening. Moreover, besides ethylene signaling, auxin signaling was also involved in banana pulp ripening by regulating cell wall and starch metabolism. Thus, the integrated analysis enabled us to get comprehensive information on the biological events that are relevant to the regulation of banana fruit ripening.

## Figures and Tables

**Table 1:** The identities of differentially expressed proteins in pulp during banana fruit ripening. Protein expression levels are represented by the column configuration, and expression levels at F1, F2, F3, and F4 are shown from left to right. Protein information is from banana genome data (http://banana-genome.cirad.fr/).

Sample Name		Protein Accumulation	Protein Name	Protein Mass (kDa)	Isoelectric Point	Pep Count	Protein Score
**Response to Stimulus (10)**							
F6		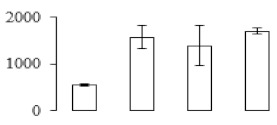	Glycine cleavage system H protein	16.89	4.99	4	142
F31		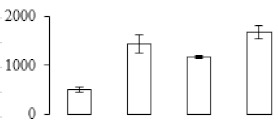	Whole genome shotgun sequence of line PN40024	14.19	4.93	14	270
F103		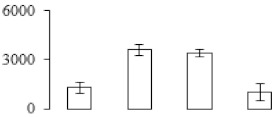	Thaumatin-like protein	20.31	4.98	6	173
F80		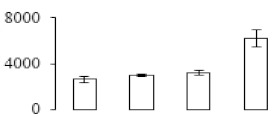	Lichenase	36.43	8.83	12	386
F25		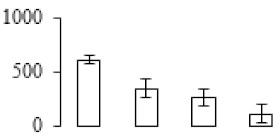	DNA-damage-repair/toleration protein DRT102	45.68	7.93	11	106
F58		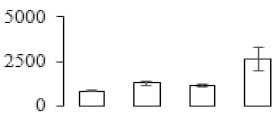	Cysteine synthase	34.05	5.27	16	304
F95		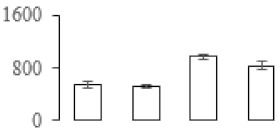	2,3-bisphosphoglycerate-independent phosphoglycerate mutase	61.21	6.05	11	109
F55		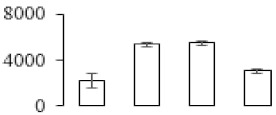	Triosephosphate isomerase	33.14	7.75	19	337
F110		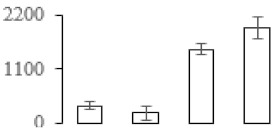	Cysteine proteinase inhibitor 12	23.08	6.66	10	159
F13		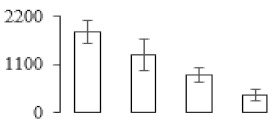	Thaumatin-like protein	20.31	4.98	5	98.2
**Oxidation-Reduction (13)**							
F32		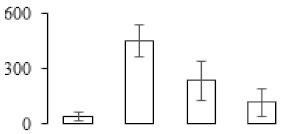	1,2-dihydroxy-3-keto-5-methylthiopentene dioxygenase 2	23.40	4.92	7	67.3
F37		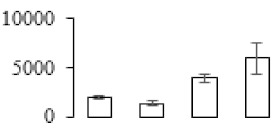	l-Ascorbate peroxidase	27.40	4.94	9	102
F43		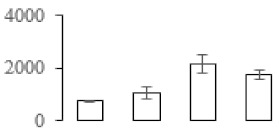	Polyphenol oxidase	62.51	6.71	19	335
F44		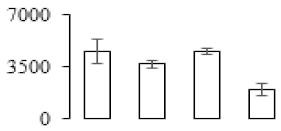	Polyphenol oxidase	62.51	6.71	21	430
F49		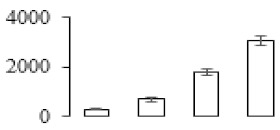	Heat shock cognate 70 kDa protein	71.30	4.83	21	216
F50		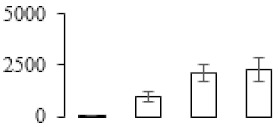	Heat shock cognate 70 kDa protein	53.44	4.81	22	146
F51		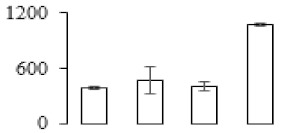	Heat shock cognate 70 kDa protein	71.30	4.83	12	59
F53		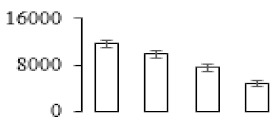	l-Ascorbate peroxidase	27.48	5.20	16	464
F56		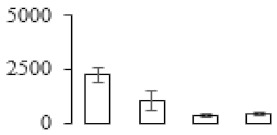	l-Ascorbate peroxidase	27.48	5.20	11	73.6
F57		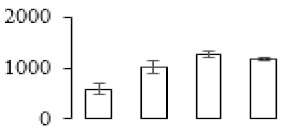	Enoyl-[acyl-carrier-protein] reductase [NADH]	40.66	8.93	9	97.5
F68		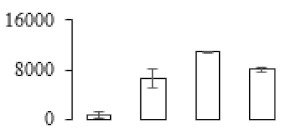	l-Ascorbate peroxidase	27.48	5.20	8	128
F70		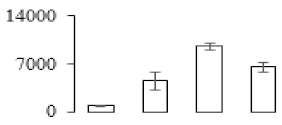	glutaredoxin	25.67	9.36	10	63.2
F45		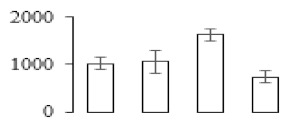	Succinyl-CoA ligase [GDP-forming] subunit beta	45.32	5.86	19	170
**Cell Wall Organization or Biogenesis (2)**							
F47	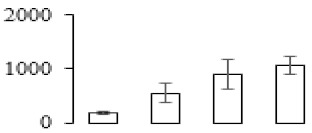		3-isopropylmalate dehydrogenase	107.41	6.27	17	62.8
F73	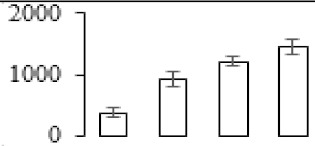		UTP–glucose-1-phosphate uridylyltransferase	51.49	5.54	10	125
**Protein Metabolic Process (9)**							
F48		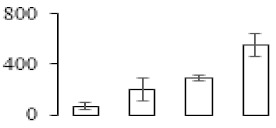	26S protease regulatory subunit 6A homolog	45.53	4.95	21	316
F106		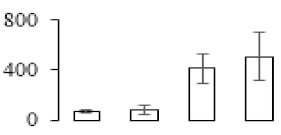	Proteasome subunit alpha type-6	27.53	6.26	15	306
F107		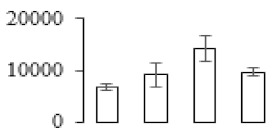	Proteasome subunit alpha type-6	27.53	6.26	11	83.2
F18		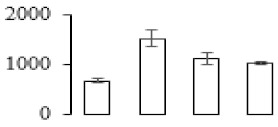	20 kDa chaperonin	27.29	7.60	7	74
F29		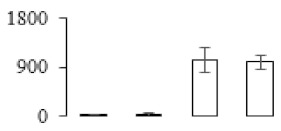	Protein disulfide-isomerase	56.62	4.51	12	97.3
F34		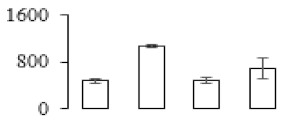	20 kDa chaperonin	27.29	7.6	11	150
F72		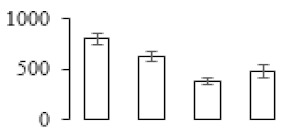	Intracellular protease 1	42.03	5.39	10	206
F96		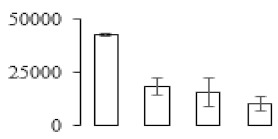	17.2 kDa class II heat shock protein	17.63	6.55	6	104
F35		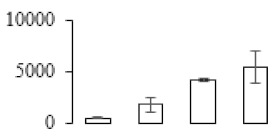	Cysteine proteinase 2	27.95	6.29	6	81.1
**Signal Transduction (6)**							
F14		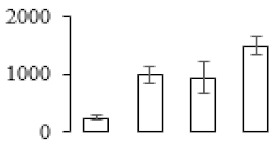	Ribulose bisphosphate carboxylase/oxygenase activase 1	52.07	5.37	16	341
F20		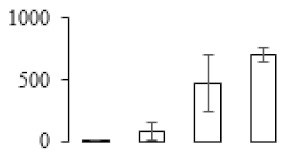	Ribulose bisphosphate carboxylase/oxygenase activase 1	52.07	5.37	12	98.2
F21		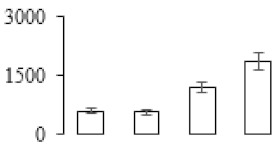	Ribulose bisphosphate carboxylase/oxygenase activase 1	52.07	5.37	14	95.8
F23		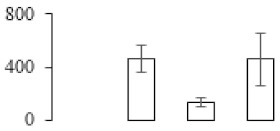	Plastid-lipid-associated protein	34.45	5.07	14	156
F87		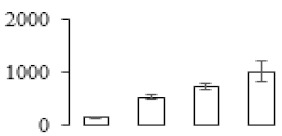	IAA-amino acid hydrolase ILR1-like 1	47.59	5.80	17	122
F93		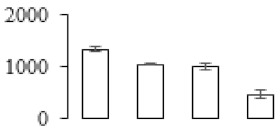	Auxin-induced protein PCNT115	43.92	8.01	15	157
**Metabolism (25)**							
F86		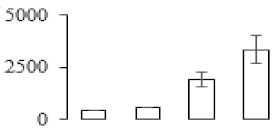	S-adenosylmethionine synthase 5	43.70	5.88	21	456
F88		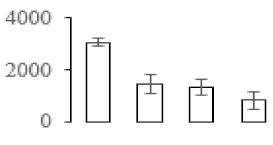	S-adenosylmethionine synthase 1	51.78	5.94	14	68.2
F22		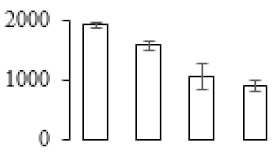	Glucose-1-phosphate adenylyltransferase large subunit 1	52.13	7.49	10	60.1
F61		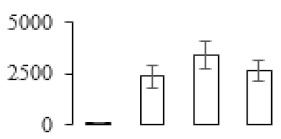	Granule-bound starch synthase 1	68.53	7.24	32	546
F64		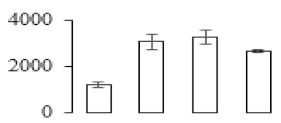	Granule-bound starch synthase 1	68.53	7.24	17	70.2
F65		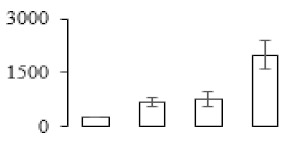	Granule-bound starch synthase 1	68.53	7.24	14	99.6
F67		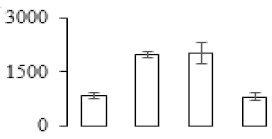	Granule-bound starch synthase 1	68.53	7.24	13	83.1
F75		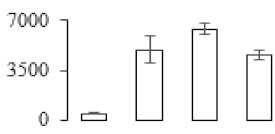	Granule-bound starch synthase 1	68.53	7.24	21	259
F84		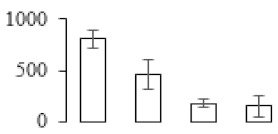	Thiazole biosynthetic enzyme	9.61	4.24	6	105
F85		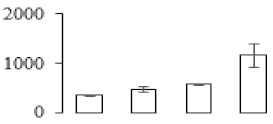	Phosphoglycerate kinase	50.16	9.23	11	239
F76		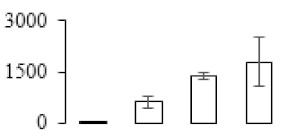	Putative Acidic endochitinase	19.57	4.86	2	72.6
F82		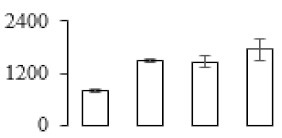	Putative Acidic endochitinase	19.57	4.86	2	111
F94		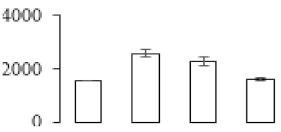	Putative Acidic endochitinase	19.57	4.86	3	66.9
F97		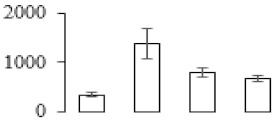	Putative Acidic endochitinase	19.57	4.86	2	153
F10		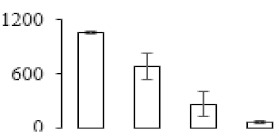	Putative Acidic endochitinase	19.57	4.86	3	148
F104		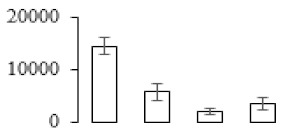	Putative Acidic endochitinase	19.57	4.86	3	121
F54		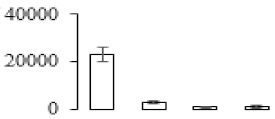	Putative Acidic endochitinase	19.57	4.86	2	113
F78		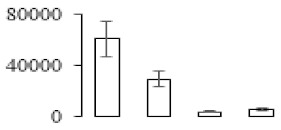	Putative Acidic endochitinase	19.57	4.86	2	179
F79		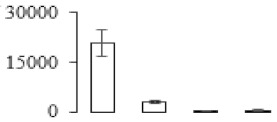	Putative Acidic endochitinase	19.57	4.86	2	115
F90		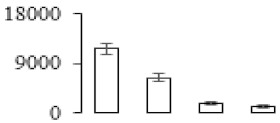	Putative Acidic endochitinase	19567.44	4.86	3	98.3
F91		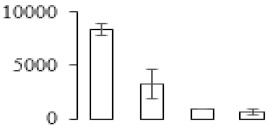	Putative Acidic endochitinase	19.57	4.86	2	112
F92		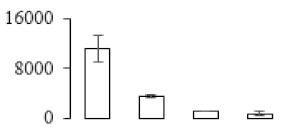	Putative Acidic endochitinase	19.57	4.86	2	82.2
F99		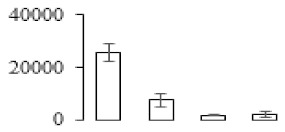	Putative Acidic endochitinase	19.57	4.86	2	108
F100		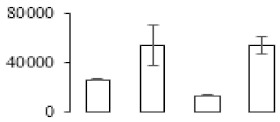	Endochitinase	34.14	6.67	5	241
F83		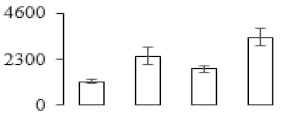	Endochitinase CH5B	36.81	8.51	1	116
**Energy (8)**							
F5		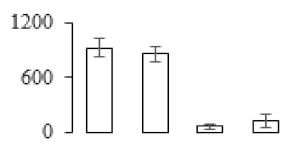	2,3-bisphosphoglycerate-dependent phosphoglycerate mutase	41.13	9.35	9	60.7
F59		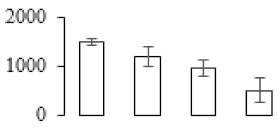	Phosphoglycerate kinase	42.52	5.09	6	93.5
F60		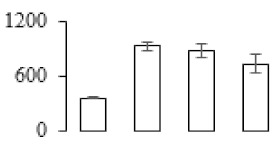	Enolase	48.25	5.74	10	128
F62		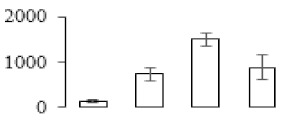	ATP synthase subunit beta	59.54	6.09	11	64.8
F81		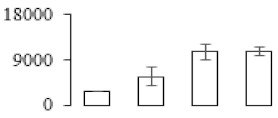	ATP synthase subunit alpha	55.80	7.02	13	69.1
F77		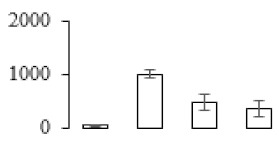	kinesin motor domain containing protein	146.25	7.36	20	68.6
F101		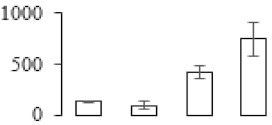	6-phosphofructokinase 2	51.86	6.23	17	135
F105		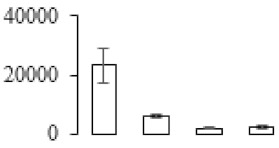	Pyrophosphate—fructose 6-phosphate 1-phosphotransferase subunit alpha	68.63	7.52	13	59.6
**Other Metabolism (11)**							
F28	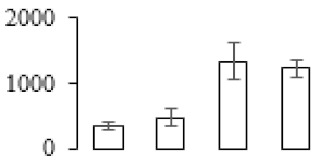		Putative Vacuolar protein sorting-associated protein 35	90.05	5.47	14	61.9
F36	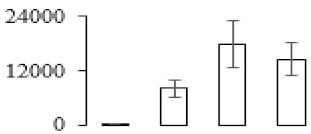		Germin-like protein 12-1	25.23	5.99	4	228
F40	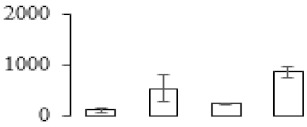		sugar transporter superfamily	68.45	8.07	11	60.8
F66	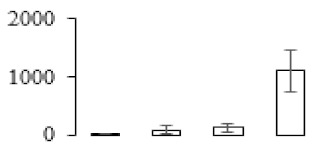		phospholipid-transporting ATPase 9	126.37	5.90	15	59
F7	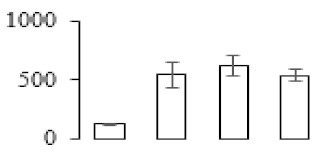		Pentatricopeptide repeat-containing protein	45. 40	8.03	11	67.1
F71	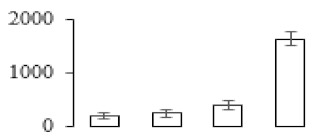		Pathogen-related protein	26.60	5.40	16	252
F8		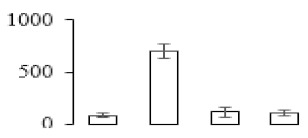	Glycine-rich RNA-binding protein 2	15.49	7.51	7	123
F12		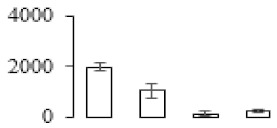	Putative disease resistance protein RGA1	113.61	7.93	18	68.8
F24		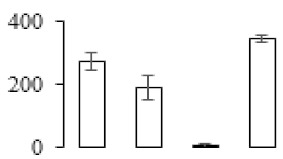	Sec-independent protein translocase protein tatA/E homolog	15.12	10.11	7	60.8
F27		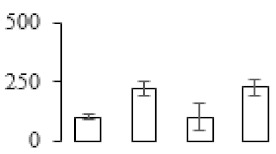	Putative DNA repair protein RAD23-3	43.27	4.54	9	85.2
F30		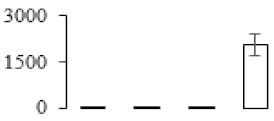	Formin-like protein 6	23.65	7.95	9	62.6

**Table 2:** Differential accumulated primary metabolites in the ripening process of banana flesh. The accumulations of different stages were indicated as the ratio compared to F1. Asterisks represent statistically significant differences (*p* < 0.05).

Compound Name	Accumulation			RT (min)
	F2/F1	F3/F1	F4/F1	
**Sugars**				
Lactulose	0.00 *	6.34	1.26	53.06
α-d-Mannopyranoside	1.21	1.69 *	3.73 *	30.69
α-l-Galactopyranose-	155.74 *	295.90 *	680.69 *	28.63
d-(+)-Turanose	4.43 *	5.69 *	2.68	52.36
d-(-)-Fructopyranose	34.35 *	83.76	174.39 *	27.11
Ribitol	1.81 *	1.71 *	1.82 *	23.04
α-d-Glucofuranosyl benzenesulfonate	0.33	1.16	0.00*	8.17
**Organic Acids**				
Butanoic acid	0.80	0.68	1.47	15.97
10,12-Tricosadiynoic acid	0.80	0.72	0.91	58.78
Propanoic acid	2.59	0.61	1.33	55.98
Ethanedioic acid	0.00*	0.00*	1.18	4.39
2-Butenedioic acid	2.27	3.77	1.57	10.01
Butanedioic acid	3.82 *	5.09	7.94 *	14.66
1,2,3-Propanetricarboxylic acidster	3.88	3.75	7.43	26.29
Benzenemethano	2.48	1.56	1.06	4.47
**Fatty Acids**				
Hexadecanoic acid	0.69	0.63	0.75	52.00
Dehydroabietic acid	0.78	0.99	2.67	49.62
Octadecanoic acid	0.81	0.95	1.19	46.68
Arachidonic acid	0.82	1.04	0.80 *	18.94
9,12,15-Octadecatrienoic acid	2.75	2.08*	1.57	48.96
Octadecanoic acid	1.52	0.99*	1.22	53.67
Hexadecanoic acid	0.69	1.14	1.88	34.71
**Alkanes**				
Trisiloxane	0.65 *	1.06 *	1.07	9.00
Silane	0.74	0.89	1.04	17.55
**Alcohols**				
6-Amino-1-hexanol	0.31	0.37	1.24 *	12.47
Myo-Inositol	2.26 *	1.95 *	3.40 *	36.47
Glycerol	1.28 *	1.33 *	1.35	7.62
Isoborneol	2.95	1.11	1.45	13.92
Borneol	0.31	0.33	2.07	30.19
D-Pinitol	1.12	1.54	1.38	27.62
**Alkali**				
Benzoylamide	0.63	1.09	0.59	23.43
5-Methanesulfonyl	1.02	1.27	1.41	31.57
**Aldehydes**				
9,12-Octadecadienal	0.43	1.20	0.77	46.26
**Others**				
4,5-Dihydrobenzo[1,2-c:3,4-c’]bis [1,2,5]oxadiazole-1,6-dioxide	3.36	4.42	0.00	6.51
1,3-Benzoxazol	1.01	1.26 *	0.99	16.22
1-[4-(2,2-Difluoroacetyl)piperazin-1-yl]-2,2-difluoroethanone	0.00 *	0.00 *	0.00 *	17.39
(Bicyclopentylidene-2-yloxy)trimethylsilane	0.00 *	0.00 *	0.00 *	29.92
10-Acetoxy-2-hydroxy	0.45	0.36	1.25	30.29
6-Dimethyl(chloromethyl)silyloxypentadecane	0.94	1.75	2.78	50.27

RT: Retention time.

## Supplementary Materials

The following are available online at https://www.mdpi.com/2218-273X/9/10/523/s1, Figure S1: The pipeline of all the analysis done in this study. Figure S2: The ultrastructure of mitochondria of banana flesh cell in different ripening stages. A: F1; B: F2; C: F3; D: F4. Figure S3: Validation of the RNA-Seq expression data using quantitative real-time PCR. Figure S4: Correlation between the differentially expressed proteins and genes in the whole library. Figure S5: Clustering of signal transduction related genes using BiNGO 2.44. Figure S6: Clustering of sugar metabolism related genes using BiNGO 2.44. Figure S7: Schematic of the cell wall metabolism pathway using the MapMan visualization platform. Figure S8: Schematic of the secondary metabolism pathway using the MapMan visualization platform. Table S1: Specific primers used in qRT-PCR. Table S2: Some differentially expressed genes mentioned in this paper.
