# Supplementary material for: Combination of Transcriptomic, Proteomic, and Metabolomic Analysis Reveals the Ripening Mechanism of Banana Pulp
Source: Biomolecules. 2019 Sep 23;9(10):523. doi: 10.3390/biom9100523 (PMC6843284; doi:10.3390/biom9100523)
Supplement: Supplementary file 1 [file biomolecules-09-00523-s001.pdf]

Supplementary Materials

Combination of Transcriptomic, Proteomic, and Metabolomic Analysis Reveals the Ripening Mechanism of Banana Pulp

Taotao Li [1,†], Ze Yun [1,†], Qixian Wu [1,2], Hongxia Qu [1], Xuewu Duan [1,*], and Yueming Jiang [1]

1 Key Laboratory of Post-Harvest Handling of Fruits, Ministry of Agriculture/Guangdong Provincial Key Laboratory of Applied Botany/Key Laboratory of Plant Resources Conservation and Sustainable Utilization, Guangzhou 510650

2 University of Chinese Academy of Sciences, Beijing 100039, P.R. China

† These authors contributed equally to this work

* Correspondence: xwduan@scbg.ac.cn

Table S1. Specific primers used in qRT-PCR.

Gene ID*	Gene name	Primer sequences (5'-3')	Product length (bp)
GSMUA_Achr11T22960_001	*Expansin-A8*	For: TACGGCATCAAGAACACGGCACTGAG<br>Rev: AGTAGTTGGTGGCGGTGACGGTGAT	145
GSMUA_Achr11T25010_001	*glycosyl transferase, family 8*	For: TCTTGATGTTAATGCGAGTGACCTGTCCAA<br>Rev: GTGCCCTTCCCTGCTACAACCAACT	102
GSMUA_Achr5T15890_001	*auxin efflux carrier component*	For: CATTCTCGGTGGCAATCTAACAGAAGGACT<br>Rev: CAATACAGCGGAGATCGTGGCAGGAA	165
GSMUA_Achr11T05430_001	*Pectinesterase 3*	For: GGAAGAGCCAGACGAACCTGATGTT<br>Rev: AGGAAGCCATTGCCGACGATAGC	133
GSMUA_Achr3T10540_001	*Putative Cytochrome P450*	For: TGTGCTTGCCAACCTCCTCTACTCCT<br>Rev: GCTTCCTCCATGCTGATGTCCTCCTTC	81
GSMUA_Achr10T12920_001	*Trans-cinnamate 4-monooxygenase*	For: GCATGGACGACCCGCTCTTCAACA<br>Rev: CCATCTTCTTCTTCTTCTCCGCCACGAA	207
GSMUA_Achr6T32320_001	*NAC domain-containing protein*	For: CATCATCGCCGAGGTGGACATCTACAAG<br>Rev: ACCAACGCCTTCTTCACTCCGATATGC	228
GSMUA_Achr6T28260_001	*Probable pectate lyase*	For: GCAGGTCACCATAGCCTTCAACCACTT<br>Rev: AGTTCCACTTCTTCCACTCGCTCTCCT	245
GSMUA_Achr8T12920_001	*Probable aquaporin TIP1-1*	For: GATCTACTGGCTCGGTCCGCTCAT<br>Rev: TCGTGGGAGTGGCTGATGAAGAAGAA	81
GSMUA_Achr5T07470_001	*Expansin-A2*	For: TGTGTGAAGAAGGGAGGCGTAAGGT<br>Rev: CAGGTACGAGTTGCTCTGCCAGTTC	180
GSMUA_Achr5T20330_001	*Peroxidase 5*	For: GCATCTTGAAGCACCGTGGTCTGT<br>Rev: AGAGTTGGTCGCCAGCAGAGTCA	95
GSMUA_Achr6T33120_001	*L-ascorbate peroxidase, cytosolic*	For: CGATAAGGCTCTCCTCACCGATCC<br>Rev: CAGCATAGTCAGCAAAGAAGGCATCC	89
GSMUA_Achr4T17840_001	*Beta-amylase 3*	For: CCGACGACAGCATTGACCTGAGTG<br>Rev: GGCACAACCTTCCTTCCACCTTCC	130
GSMUA_Achr5T21880_001	*Probable xyloglucan endotransglucosylase/hydrolase*	For: CCTTTCCCAACAGCCAGCCCAT<br>Rev: AAGCACACTCGGAGGTAGCAGAC	174
GSMUA_Achr11T12650_001	*Putative Ethylene-responsive transcription factor*	For: AGCTCAACTTCCCGGAGTCGGTAGA<br>Rev: TCCGCCAGCTCCAACCACATCTT	208
GSMUA_Achr4T31990_001	*Auxin-responsive family protein*	For: GCCACCGATTTGAAGCCGTCTAC<br>Rev: ACCTCGAACCGCTCCATCTCCT	208
GSMUA_Achr7T26370_001	*Probable indole-3-acetic acid-amido synthetase*	For: ATCCTCTCCGCCCATCCCATCTCT<br>Rev: GGCTCTGGAAGTGCTCGCTCTTGTA	271
GSMUA_Achr4T29150_001	*1-aminocyclopropane-1-carboxylate synthase*	For: TCACATCGCCTACAGCCTCTCCAAG<br>Rev: AACTCGTGGTGAACTCCTCGTCTCC	182
GSMUA_Achr3T20240_001	*Actin1*	For: TGGTATGGAAGCCGCTGGTA<br>Rev: TCTGCTGGAATGTGCTGAGG	236

* The ID was obtained from *Banana* genome in http://banana-genome.cirad.fr/home.

Table 2. Some differentially expressed genes mentioned in this paper. #N/A: Not significantly differentially expressed.

GeneID	log2(F2/F1)	log2(F3/F1)	log2(F4/F1)	Description
Sugar metabolism				
GSMUA_Achr5T28150_001	#N/A	#N/A	11.889323	Hypothetical protein
GSMUA_Achr9T16670_001	#N/A	#N/A	11.158097	Polygalacturonase
GSMUA_Achr4T20850_001	#N/A	#N/A	11.153666	Putative Phosphoenolpyruvate carboxykinase [ATP]
GSMUA_Achr5T28160_001	#N/A	#N/A	10.948538	Hypothetical protein
GSMUA_Achr4T28890_001	#N/A	#N/A	5.7833021	Pyruvate kinase isozyme G
GSMUA_Achr2T14330_001	#N/A	11.274208	#N/A	Putative Polygalacturonase At1g48100
GSMUA_AchrUn_randomT27170_001	#N/A	11.009196	#N/A	Sugar transport protein 13
GSMUA_Achr1T00870_001	#N/A	10.9725	10.57192	Putative 21 kDa protein
GSMUA_Achr7T15620_001	#N/A	8.0811427	7.5249613	Pectinesterase/pectinesterase inhibitor PPE8B
GSMUA_Achr11T09430_001	#N/A	6.910651	#N/A	Probable NAD(P)H-dependent oxidoreductase 1
GSMUA_Achr6T05900_001	#N/A	5.8032888	6.8782159	Putative 21 kDa protein
GSMUA_Achr5T28140_001	#N/A	5.5809782	10.329613	Pyruvate decarboxylase isozyme 2
GSMUA_Achr7T18470_001	#N/A	5.3413789	#N/A	Probable galacturonosyltransferase-like 9
GSMUA_Achr7T26570_001	#N/A	5.2180913	4.933326	Hypothetical protein
GSMUA_Achr4T16250_001	#N/A	5.1970539	#N/A	Fructokinase-1
GSMUA_Achr1T23960_001	#N/A	5.1577286	#N/A	Cellulose synthase-like protein D2
GSMUA_Achr3T05750_001	#N/A	5.089126	#N/A	pectinesterase/pectinesterase inhibitor 12
GSMUA_Achr7T16250_001	#N/A	4.9752569	#N/A	UDP-glucuronate 4-epimerase 1
GSMUA_Achr6T11090_001	#N/A	4.9556703	#N/A	UDP-glucose 6-dehydrogenase
GSMUA_Achr3T03290_001	#N/A	4.862395	5.3423045	Soluble starch synthase 1
GSMUA_Achr5T07390_001	#N/A	4.3722631	#N/A	Pyruvate decarboxylase isozyme 2
GSMUA_Achr3T10110_001	#N/A	4.3272963	#N/A	Pyruvate decarboxylase isozyme 2
GSMUA_Achr10T18280_001	#N/A	4.0475892	#N/A	UDP-glucose 6-dehydrogenase
GSMUA_Achr7T11480_001	#N/A	3.7920904	#N/A	Pectinesterase 3
GSMUA_Achr8T04140_001	#N/A	3.6697306	#N/A	Alpha-amylase
GSMUA_Achr7T26580_001	#N/A	3.3392906	#N/A	UDP-glucose 6-dehydrogenase
GSMUA_Achr6T27190_001	#N/A	-4.174692	#N/A	Glucose-1-phosphate adenylyltransferase large subunit 2,
GSMUA_Achr3T23340_001	#N/A	-4.270252	#N/A	Probable alpha-glucosidase
GSMUA_Achr3T29530_001	#N/A	-5.034086	#N/A	Beta-glucosidase 1
GSMUA_Achr3T23330_001	#N/A	-5.447644	#N/A	Hypothetical protein
GSMUA_Achr10T00650_001	8.0489263	#N/A	#N/A	Fructose-1,6-bisphosphatase
GSMUA_Achr6T17900_001	7.8202119	#N/A	5.537678	Fructose-bisphosphate aldolase
GSMUA_Achr8T11740_001	6.1151529	7.4362729	6.9971128	Aldehyde dehydrogenase family 3 member F1
GSMUA_Achr8T17280_001	5.4310798	#N/A	#N/A	Fructose-bisphosphate aldolase
GSMUA_Achr7T19810_001	4.9188828	#N/A	#N/A	Sucrose synthase 1
GSMUA_Achr5T08790_001	4.8843495	#N/A	#N/A	Putative Lysosomal beta glucosidase
GSMUA_Achr4T14320_001	4.1942108	5.0695261	#N/A	UDP-glucose 6-dehydrogenase
GSMUA_Achr4T17840_001	3.2512846	4.7044814	4.8279554	Beta-amylase 3
GSMUA_Achr8T18810_001	-3.189724	-4.678706	#N/A	Phosphoenolpyruvate carboxykinase [ATP]
GSMUA_Achr8T08790_001	-3.400789	-5.865745	#N/A	UDP-glucuronate 4-epimerase 6
GSMUA_Achr11T05430_001	-3.835821	-5.706748	-5.044671	Pectinesterase 3
GSMUA_Achr11T25010_001	-4.150236	-6.983959	-4.829516	glycosyl transferase, family 8, putative
GSMUA_Achr5T28150_001	#N/A	#N/A	11.889323	Hypothetical protein
GSMUA_Achr9T16670_001	#N/A	#N/A	11.158097	Polygalacturonase
GSMUA_Achr4T20850_001	#N/A	#N/A	11.153666	Putative Phosphoenolpyruvate carboxykinase [ATP]
GSMUA_Achr5T28160_001	#N/A	#N/A	10.948538	Hypothetical protein
GSMUA_Achr4T28890_001	#N/A	#N/A	5.7833021	Pyruvate kinase isozyme G
GSMUA_Achr2T14330_001	#N/A	11.274208	#N/A	Putative Polygalacturonase
GSMUA_AchrUn_randomT27170_001	#N/A	11.009196	#N/A	Sugar transport protein 13
GSMUA_Achr1T00870_001	#N/A	10.9725	10.57192	Putative 21 kDa protein
GSMUA_Achr7T15620_001	#N/A	8.0811427	7.5249613	Pectinesterase/pectinesterase inhibitor PPE8B
GSMUA_Achr11T09430_001	#N/A	6.910651	#N/A	Probable NAD(P)H-dependent oxidoreductase 1
GSMUA_Achr6T05900_001	#N/A	5.8032888	6.8782159	Putative 21 kDa protein
GSMUA_Achr5T28140_001	#N/A	5.5809782	10.329613	Pyruvate decarboxylase isozyme 2
GSMUA_Achr7T18470_001	#N/A	5.3413789	#N/A	Probable galacturonosyltransferase-like 9
GSMUA_Achr7T26570_001	#N/A	5.2180913	4.933326	Hypothetical protein
GSMUA_Achr4T16250_001	#N/A	5.1970539	#N/A	Fructokinase-1
GSMUA_Achr1T23960_001	#N/A	5.1577286	#N/A	Cellulose synthase-like protein D2
GSMUA_Achr3T05750_001	#N/A	5.089126	#N/A	pectinesterase/pectinesterase inhibitor 12
GSMUA_Achr7T16250_001	#N/A	4.9752569	#N/A	UDP-glucuronate 4-epimerase 1
GSMUA_Achr6T11090_001	#N/A	4.9556703	#N/A	UDP-glucose 6-dehydrogenase
GSMUA_Achr3T03290_001	#N/A	4.862395	5.3423045	Soluble starch synthase 1, chloroplastic/amyloplastic
GSMUA_Achr5T07390_001	#N/A	4.3722631	#N/A	Pyruvate decarboxylase isozyme 2
GSMUA_Achr3T10110_001	#N/A	4.3272963	#N/A	Pyruvate decarboxylase isozyme 2
GSMUA_Achr10T18280_001	#N/A	4.0475892	#N/A	UDP-glucose 6-dehydrogenase
GSMUA_Achr7T11480_001	#N/A	3.7920904	#N/A	Pectinesterase 3
GSMUA_Achr8T04140_001	#N/A	3.6697306	#N/A	Alpha-amylase
GSMUA_Achr7T26580_001	#N/A	3.3392906	#N/A	UDP-glucose 6-dehydrogenase

GSMUA_Achr6T27190_001	#N/A	-4.174692	#N/A	Glucose-1-phosphate adenylyltransferase large subunit 2
GSMUA_Achr3T23340_001	#N/A	-4.270252	#N/A	Probable alpha-glucosidase
GSMUA_Achr3T29530_001	#N/A	-5.034086	#N/A	Beta-glucosidase 1
GSMUA_Achr3T23330_001	#N/A	-5.447644	#N/A	Hypothetical protein
GSMUA_Achr10T00650_001	8.0489263	#N/A	#N/A	Fructose-1,6-bisphosphatase
GSMUA_Achr6T17900_001	7.8202119	#N/A	5.537678	Fructose-bisphosphate aldolase
GSMUA_Achr8T11740_001	6.1151529	7.4362729	6.9971128	Aldehyde dehydrogenase family 3 member F1
GSMUA_Achr8T17280_001	5.4310798	#N/A	#N/A	Fructose-bisphosphate aldolase
GSMUA_Achr7T19810_001	4.9188828	#N/A	#N/A	Sucrose synthase 1
GSMUA_Achr5T08790_001	4.8843495	#N/A	#N/A	Putative Lysosomal beta glucosidase
GSMUA_Achr4T14320_001	4.1942108	5.0695261	#N/A	UDP-glucose 6-dehydrogenase
GSMUA_Achr4T17840_001	3.2512846	4.7044814	4.8279554	Beta-amylase 3, chloroplastic
GSMUA_Achr8T18810_001	-3.189724	-4.678706	#N/A	Phosphoenolpyruvate carboxykinase [ATP]
GSMUA_Achr8T08790_001	-3.400789	-5.865745	#N/A	UDP-glucuronate 4-epimerase 6
GSMUA_Achr11T05430_001	-3.835821	-5.706748	-5.044671	Pectinesterase 3
GSMUA_Achr11T25010_001	-4.150236	-6.983959	-4.829516	glycosyl transferase, family 8
secondary metabolism related genes				
GSMUA_Achr7T20440_001	3.8334438	#N/A	#N/A	Serine--glyoxylate aminotransferase
GSMUA_Achr10T18000_001	7.398088	#N/A	#N/A	3-ketoacyl-CoA synthase 6
GSMUA_Achr7T19200_001	8.2319678	#N/A	#N/A	Putative (+)-delta-cadinene synthase isozyme A
GSMUA_Achr6T17900_001	7.8202119	#N/A	5.537678	Fructose-bisphosphate aldolase
GSMUA_Achr3T02180_001	10.438443	#N/A	#N/A	3-ketoacyl-CoA synthase 6
GSMUA_Achr8T10230_001	3.8635322	#N/A	#N/A	UDP-glucuronic acid decarboxylase 1
GSMUA_AchrUn_randomT27450_001	6.8909264	#N/A	#N/A	Putative (+)-delta-cadinene synthase isozyme A
GSMUA_Achr9T00170_001	4.1574005	5.7016985	4.8250595	UDP-glucose 4-epimerase GEPI48
GSMUA_Achr6T30280_001	8.2122544	#N/A	#N/A	Putative Acidic mammalian chitinase
GSMUA_Achr5T08790_001	4.8843495	#N/A	#N/A	Putative Lysosomal beta glucosidase
GSMUA_Achr2T06930_001	3.2766542	#N/A	#N/A	Putative Haloacid dehalogenase-like hydrolase domain-containing protein 3
GSMUA_Achr2T14310_001	4.7772217	6.1834683	#N/A	Chalcone synthase E
GSMUA_AchrUn_randomT13750_001	11.183816	#N/A	#N/A	Putative (+)-delta-cadinene synthase
GSMUA_Achr9T29540_001	6.3137197	#N/A	#N/A	Putative Peroxidase 12
GSMUA_Achr7T19220_001	11.239928	#N/A	#N/A	Putative (+)-delta-cadinene synthase isozyme A
GSMUA_AchrUn_randomT23970_001	6.7205808	9.838444	11.633266	Cytochrome P450-1
GSMUA_Achr7T11330_001	11.059776	#N/A	#N/A	Peroxidase 15
GSMUA_Achr10T12080_001	3.8571695	6.2178166	#N/A	Obtusifoliol 14-alpha demethylase
GSMUA_AchrUn_randomT20470_001	8.0270864	#N/A	#N/A	Putative (+)-delta-cadinene synthase isozyme A
GSMUA_Achr3T17110_001	10.235707	#N/A	#N/A	Putative Estradiol 17-beta-dehydrogenase 12
GSMUA_AchrUn_randomT25250_001	5.0107717	5.0249177	#N/A	Putative Polyneuridine-aldehyde esterase
GSMUA_Achr7T19230_001	7.1461341	#N/A	#N/A	Putative Germacrene-D synthase
GSMUA_Achr8T17280_001	5.4310798	#N/A	#N/A	Fructose-bisphosphate aldolase
GSMUA_Achr4T31780_001	10.572519	#N/A	#N/A	Uncharacterized oxidoreductase
GSMUA_Achr7T07880_001	6.6074744	4.3293196	#N/A	Probable peroxisomal (S)-2-hydroxy-acid oxidase 2
GSMUA_Achr5T20330_001	4.9459265	4.6176329	#N/A	Peroxidase 5
GSMUA_Achr1T06310_001	12.19999	#N/A	10.86604	Glycine cleavage system H protein
GSMUA_AchrUn_randomT18440_001	12.116833	#N/A	#N/A	Putative Germacrene A synthase long form
GSMUA_Achr11T15080_001	4.7505775	#N/A	#N/A	BTBZ2 - Bric-a-Brac, Tramtrack, and Broad Complex BTB domain with TAZ zinc finger and Calmodulin-binding domains,
GSMUA_Achr6T14620_001	3.5061891	5.4906648	#N/A	Putative Peroxidase 52
GSMUA_Achr9T13520_001	10.167764	8.9080272	#N/A	Protein WAX2
GSMUA_Achr6T21330_001	8.7710123	#N/A	#N/A	Endochitinase PR4
GSMUA_AchrUn_randomT13780_001	12.731886	#N/A	#N/A	Hypothetical protein
GSMUA_Achr7T19240_001	10.650366	#N/A	#N/A	Putative (+)-delta-cadinene synthase isozyme A
GSMUA_Achr3T02010_001	7.513584	6.9412215	6.7881713	3-ketoacyl-CoA synthase 10
GSMUA_Achr7T19260_001	5.9339962	#N/A	#N/A	Vetispiradiene synthase 1
GSMUA_Achr10T00650_001	8.0489263	#N/A	#N/A	Fructose-1,6-bisphosphatase
GSMUA_Achr11T11790_001	5.6182033	7.3003911	7.6773237	Polyneuridine-aldehyde esterase
GSMUA_Achr1T04740_001	12.173085	#N/A	10.706987	3-ketoacyl-CoA synthase 17
GSMUA_Achr8T18810_001	-3.189724	-4.678706	#N/A	Phosphoenolpyruvate carboxykinase [ATP]
GSMUA_Achr5T11300_001	6.8071781	#N/A	#N/A	3-ketoacyl-CoA synthase 1
GSMUA_Achr11T23450_001	3.4573578	4.255342	#N/A	Probable 4-coumarate--CoA ligase 2
GSMUA_Achr4T29150_001	10.093263	11.255807	8.0855062	1-aminocyclopropane-1-carboxylate synthase
GSMUA_Achr7T19250_001	7.9395639	#N/A	#N/A	Putative (+)-delta-cadinene synthase isozyme A
GSMUA_Achr2T11090_001	6.2803898	#N/A	#N/A	Putative GDSL esterase/lipase
GSMUA_Achr4T07810_001	11.639771	#N/A	#N/A	hydrolase, alpha/beta fold family domain containing protein
GSMUA_Achr3T10540_001	10.418868	13.557513	15.268244	Putative Cytochrome P450 71A9

GSMUA_Achr7T19190_001	6.1307757	#N/A	#N/A	Putative (+)-delta-cadinene synthase isozyme XC14
GSMUA_Achr1T04730_001	11.951515	#N/A	#N/A	Putative 3-ketoacyl-CoA synthase 11
GSMUA_Achr11T25010_001	-4.150236	-6.983959	-4.829516	glycosyl transferase, family 8
GSMUA_Achr5T03490_001	12.838817	11.408783	12.709871	Putative Dihydroflavonol-4-reductase
GSMUA_Achr3T25050_001	4.6789856	#N/A	#N/A	Magnesium-protoporphyrin IX monomethyl ester [oxidative] cyclase
GSMUA_Achr2T03950_001	6.1677994	8.8235155	9.4857735	Putative peroxisomal-coenzyme A synthetase
GSMUA_Achr11T24060_001	10.089986	10.264349	#N/A	Tropinone reductase homolog
GSMUA_AchrUn_randomT13720_001	5.7119211	#N/A	#N/A	Putative (+)-delta-cadinene synthase
GSMUA_Achr3T07030_001	11.027406	#N/A	#N/A	Flavonol synthase/flavanone 3-hydroxylase
GSMUA_AchrUn_randomT27430_001	9.5325856	#N/A	#N/A	Putative Casbene synthase, chloroplastic
GSMUA_Achr11T01900_001	7.0650247	#N/A	#N/A	Protochlorophyllide reductase B
GSMUA_Achr5T11310_001	11.097794	#N/A	#N/A	Putative 3-ketoacyl-CoA synthase 1
GSMUA_Achr10T00370_001	4.2126663	#N/A	#N/A	Magnesium-protoporphyrin IX monomethyl ester [oxidative] cyclase
GSMUA_Achr6T00040_001	6.00509	#N/A	#N/A	Cytochrome P450 85A1
GSMUA_Achr10T12920_001	3.4141129	5.1445819	4.7591894	Trans-cinnamate 4-monooxygenase
GSMUA_Achr4T14320_001	4.1942108	5.0695261	#N/A	UDP-glucose 6-dehydrogenase
GSMUA_Achr2T21590_001	8.3669377	7.3656625	7.8035685	3-ketoacyl-CoA synthase 6
GSMUA_Achr8T11740_001	6.1151529	7.4362729	6.9971128	Aldehyde dehydrogenase family 3 member F1
GSMUA_Achr6T00870_001	#N/A	7.0767727	#N/A	1-aminocyclopropane-1-carboxylate oxidase
GSMUA_AchrUn_randomT25240_001	#N/A	3.9302259	#N/A	Polyneuridine-aldehyde esterase
GSMUA_AchrUn_randomT23850_001	#N/A	12.089683	12.983799	Hypothetical protein
GSMUA_Achr5T09690_001	#N/A	10.406254	#N/A	Putative 1-aminocyclopropane-1-carboxylate oxidase
GSMUA_Achr8T19570_001	#N/A	13.468699	#N/A	Putative BAHD acyltransferase DCR
GSMUA_Achr5T03950_001	#N/A	10.416196	#N/A	Phenylalanine ammonia-lyase 1
GSMUA_Achr11T21810_001	#N/A	3.7874857	4.6638635	Cytochrome P450 84A1
GSMUA_Achr11T05530_001	#N/A	4.7792368	4.9464821	NAD dependent epimerase/dehydratase family domain containing protein
GSMUA_Achr4T10640_001	#N/A	10.591419	#N/A	Dihydroflavonol-4-reductase
GSMUA_Achr5T07390_001	#N/A	4.3722631	#N/A	Pyruvate decarboxylase isozyme 2
GSMUA_Achr6T36400_001	#N/A	3.7657075	#N/A	Caffeoyl-CoA O-methyltransferase
GSMUA_AchrUn_randomT08000_001	#N/A	13.610396	#N/A	Putative Uncharacterized acetyltransferase
GSMUA_Achr5T28200_001	#N/A	-7.599928	#N/A	Asparagine synthetase [glutamine-hydrolyzing]
GSMUA_Achr7T27090_001	#N/A	3.3091626	#N/A	Cycloartenol-C-24-methyltransferase 1
GSMUA_Achr11T26490_001	#N/A	-4.516669	#N/A	Putative Cytochrome P450 71D7
GSMUA_Achr3T29530_001	#N/A	-5.034086	#N/A	Beta-glucosidase 1
GSMUA_Achr7T08410_001	#N/A	3.7353437	#N/A	3-ketoacyl-CoA synthase 11
GSMUA_AchrUn_randomT26960_001	#N/A	7.8808379	#N/A	1-aminocyclopropane-1-carboxylate oxidase
GSMUA_Achr3T10110_001	#N/A	4.3272963	#N/A	Pyruvate decarboxylase isozyme 2
GSMUA_Achr5T04550_001	#N/A	3.6853446	#N/A	Putative Anthranilate N-benzoyltransferase protein 1
GSMUA_AchrUn_randomT28250_001	#N/A	7.1334032	8.4150617	Cytochrome P450 71A1
GSMUA_Achr4T25540_001	#N/A	8.1334495	7.4185955	24-methylenesterol C-methyltransferase 2
GSMUA_Achr5T08490_001	#N/A	10.837769	11.173753	Putative Protein SRG1
GSMUA_Achr5T28140_001	#N/A	5.5809782	10.329613	Pyruvate decarboxylase isozyme 2
GSMUA_Achr10T14220_001	#N/A	15.324721	11.446926	Peroxidase 4
GSMUA_Achr6T07190_001	#N/A	11.741684	12.290913	Putative 3-hydroxybenzoate 6-hydroxylase 1
GSMUA_Achr7T02130_001	#N/A	10.487155	#N/A	S-adenosylmethionine synthase
GSMUA_Achr1T21750_001	#N/A	3.2042354	#N/A	cystathionine gamma-synthase
GSMUA_Achr11T09320_001	#N/A	-3.468818	#N/A	Putative Adipocyte plasma membrane-associated protein
GSMUA_Achr6T21530_001	#N/A	6.1187807	#N/A	Peroxidase 52
GSMUA_Achr10T19150_001	#N/A	4.780451	#N/A	Putative Cytokinin-O-glucosyltransferase 2
GSMUA_Achr9T21400_001	#N/A	5.266843	5.4311627	Hypothetical protein
GSMUA_Achr10T27850_001	#N/A	5.7362091	#N/A	Peroxidase 52
GSMUA_Achr1T01400_001	#N/A	3.6520491	5.3641495	Putative O-methyltransferase ZRP4
GSMUA_Achr9T10050_001	#N/A	-6.9825	#N/A	Phytoene synthase, chloroplastic
GSMUA_Achr7T10870_001	#N/A	3.4377689	#N/A	Probable chlorophyll(ide) b reductase NYC1
GSMUA_Achr9T27540_001	#N/A	-5.178889	#N/A	Shikimate kinase
GSMUA_Achr10T18280_001	#N/A	4.0475892	#N/A	UDP-glucose 6-dehydrogenase
GSMUA_Achr7T26570_001	#N/A	5.2180913	4.933326	Hypothetical protein
GSMUA_Achr5T04080_001	#N/A	12.58443	#N/A	Leucoanthocyanidin dioxygenase
GSMUA_AchrUn_randomT29200_001	#N/A	9.9410335	10.335514	Putative Cytokinin-O-glucosyltransferase 2
GSMUA_Achr7T26580_001	#N/A	3.3392906	#N/A	UDP-glucose 6-dehydrogenase
GSMUA_Achr10T30380_001	#N/A	-3.907197	-5.555834	Phospho-2-dehydro-3-deoxyheptonate aldolase 1
GSMUA_Achr11T09430_001	#N/A	6.910651	#N/A	Probable NAD(P)H-dependent oxidoreductase 1
GSMUA_Achr7T18470_001	#N/A	5.3413789	#N/A	Probable galacturonosyltransferase-like 9
GSMUA_Achr5T25040_001	#N/A	11.006096	#N/A	Peroxidase 55

GSMUA_AchrUn_randomT27170_001	#N/A	11.009196	#N/A	Sugar transport protein 13
GSMUA_Achr10T12200_001	#N/A	10.990642	10.287527	Chalcone synthase
GSMUA_Achr5T18560_001	#N/A	3.5787827	#N/A	Phenylalanine ammonia-lyase 1
GSMUA_Achr6T11090_001	#N/A	4.9556703	#N/A	UDP-glucose 6-dehydrogenase
GSMUA_Achr6T19880_001	#N/A	3.2380853	#N/A	Probable peroxisomal (S)-2-hydroxy-acid oxidase 2
GSMUA_Achr5T11010_001	#N/A	11.137891	12.948054	Putative Probable gibberellin receptor GID1L2
GSMUA_Achr5T03950_001	#N/A	10.416196	#N/A	Phenylalanine ammonia-lyase 1
GSMUA_Achr5T18560_001	#N/A	3.5787827	#N/A	Phenylalanine ammonia-lyase 1
GSMUA_Achr2T22970_001	#N/A	#N/A	6.7241153	Chalcone synthase
GSMUA_AchrUn_randomT26440_001	#N/A	#N/A	7.0905947	Putative Protein SRG1
GSMUA_Achr5T28150_001	#N/A	#N/A	11.889323	Hypothetical protein
GSMUA_Achr10T18200_001	#N/A	#N/A	-6.714387	Putative Isoflavone 2'-hydroxylase
GSMUA_Achr7T26390_001	#N/A	#N/A	11.248721	Putative Isoflavone 2'-hydroxylase
GSMUA_Achr11T04490_001	#N/A	#N/A	11.23298	Putative S-norcoclaurine synthase 1
GSMUA_Achr2T11130_001	#N/A	#N/A	4.8173377	Putative GDSL esterase/lipase
GSMUA_Achr4T28890_001	#N/A	#N/A	5.7833021	Pyruvate kinase isozyme G, chloroplastic
GSMUA_Achr4T20850_001	#N/A	#N/A	11.153666	Putative Phosphoenolpyruvate carboxykinase [ATP]
GSMUA_Achr5T04570_001	#N/A	#N/A	7.499047	tropinone reductase 2
GSMUA_Achr5T29600_001	#N/A	#N/A	10.786115	Peroxidase 4
GSMUA_Achr5T17150_001	#N/A	#N/A	7.501847	Hypothetical protein
GSMUA_AchrUn_randomT16580_001	#N/A	#N/A	10.467915	Putative uncharacterized protein
GSMUA_Achr1T07320_001	#N/A	#N/A	10.563969	Endochitinase
GSMUA_Achr3T02950_001	#N/A	#N/A	6.6242307	Peroxidase 12
GSMUA_Achr2T11160_001	#N/A	#N/A	5.6005034	Putative GDSL esterase/lipase
GSMUA_Achr5T28160_001	#N/A	#N/A	10.948538	Hypothetical protein
GSMUA_Achr3T16520_001	#N/A	#N/A	9.4485185	Peroxidase 43
GSMUA_Achr9T03440_001	#N/A	#N/A	7.2760967	Tropinone reductase homolog
GSMUA_Achr9T00170_001	4.1574005	5.7016985	4.8250595	UDP-glucose 4-epimerase GEPI48
GSMUA_AchrUn_randomT23970_001	6.7205808	9.838444	11.633266	Cytochrome P450-1
GSMUA_Achr3T02010_001	7.513584	6.9412215	6.7881713	3-ketoacyl-CoA synthase 10
GSMUA_Achr11T11790_001	5.6182033	7.3003911	7.6773237	Putative Polyneuridine-aldehyde esterase
GSMUA_Achr4T29150_001	10.093263	11.255807	8.0855062	1-aminocyclopropane-1-carboxylate synthase
GSMUA_Achr3T10540_001	10.418868	13.557513	15.268244	Putative Cytochrome P450 71A9
GSMUA_Achr11T25010_001	-4.150236	-6.983959	-4.829516	glycosyl transferase, family 8, putative, expressed
GSMUA_Achr5T03490_001	12.838817	11.408783	12.709871	Putative Dihydroflavonol-4-reductase
GSMUA_Achr2T03950_001	6.1677994	8.8235155	9.4857735	Putative peroxisomal-coenzyme A synthetase
GSMUA_Achr10T12920_001	3.4141129	5.1445819	4.7591894	Trans-cinnamate 4-monooxygenase
GSMUA_Achr2T21590_001	8.3669377	7.3656625	7.8035685	3-ketoacyl-CoA synthase 6
GSMUA_Achr8T11740_001	6.1151529	7.4362729	6.9971128	Aldehyde dehydrogenase family 3 member F1
oxidation-reduction				
GSMUA_Achr7T26390_001	#N/A	#N/A	11.248721	Putative Isoflavone 2'-hydroxylase
GSMUA_Achr5T29600_001	#N/A	#N/A	10.786115	Peroxidase 4
GSMUA_Achr6T01210_001	#N/A	#N/A	10.321576	Acyl-[acyl-carrier-protein] desaturase, chloroplastic
GSMUA_Achr3T16520_001	#N/A	#N/A	9.4485185	Peroxidase 43
GSMUA_Achr5T14190_001	#N/A	#N/A	7.748746	Omega-3 fatty acid desaturase
GSMUA_Achr9T03440_001	#N/A	#N/A	7.2760967	Tropinone reductase homolog
GSMUA_Achr10T14220_001	#N/A	15.324721	11.446926	Peroxidase 4
GSMUA_Achr4T15110_001	#N/A	12.038279	#N/A	Gibberellin 2-beta-dioxygenase 1
GSMUA_Achr6T07190_001	#N/A	11.741684	12.290913	Putative 3-hydroxybenzoate 6-hydroxylase 1
GSMUA_Achr5T25040_001	#N/A	11.006096	#N/A	Peroxidase 55
GSMUA_Achr6T04470_001	#N/A	10.911212	#N/A	oxidoreductase, putative
GSMUA_Achr9T06460_001	#N/A	10.473573	#N/A	Gibberellin 2-beta-dioxygenase
GSMUA_Achr6T35880_001	#N/A	10.365646	#N/A	Putative 2-aminoethanethiol dioxygenase
GSMUA_Achr8T20430_001	#N/A	9.9992879	10.312818	Short-chain type dehydrogenase/reductase
GSMUA_Achr5T26970_001	#N/A	8.0683636	#N/A	Respiratory burst oxidase homolog protein B
GSMUA_AchrUn_randomT28250_001	#N/A	7.1334032	8.4150617	Cytochrome P450 71A1
GSMUA_Achr4T18430_001	#N/A	6.9403136	#N/A	Abscisic acid 8'-hydroxylase 1
GSMUA_Achr11T09430_001	#N/A	6.910651	#N/A	Probable NAD(P)H-dependent oxidoreductase 1
GSMUA_Achr11T23780_001	#N/A	6.1878576	5.9779668	Ferritin-3, chloroplastic
GSMUA_Achr6T21530_001	#N/A	6.1187807	#N/A	Peroxidase 52
GSMUA_Achr10T27850_001	#N/A	5.7362091	#N/A	Peroxidase 52
GSMUA_Achr2T08400_001	#N/A	5.3308747	#N/A	Putative 2-aminoethanethiol dioxygenase
GSMUA_Achr8T32640_001	#N/A	5.2396533	#N/A	Acyl-[acyl-carrier-protein] desaturase, chloroplastic
GSMUA_Achr6T27140_001	#N/A	4.4052597	#N/A	siroheme synthase, putative
GSMUA_Achr4T08230_001	#N/A	4.1392846	4.7708565	Inositol oxygenase 1
GSMUA_Achr10T18280_001	#N/A	4.0475892	#N/A	UDP-glucose 6-dehydrogenase
GSMUA_Achr7T10870_001	#N/A	3.4377689	#N/A	chlorophyll(ide) b reductase NYC1
GSMUA_Achr7T26580_001	#N/A	3.3392906	#N/A	UDP-glucose 6-dehydrogenase
GSMUA_Achr4T02230_001	#N/A	3.3232868	#N/A	Putative Apoptosis-inducing factor homolog B
GSMUA_Achr6T33120_001	#N/A	-3.195639	-4.337421	L-ascorbate peroxidase, cytosolic

GSMUA_Achr8T13460_001	11.961653	#N/A	#N/A	Ferredoxin--NADP reductase, leaf isozyme
GSMUA_Achr4T07780_001	11.889718	#N/A	#N/A	Quinone oxidoreductase-like protein
GSMUA_Achr7T24700_001	11.718745	10.018997	11.572278	Putative Uncharacterized hydroxylase C887.15c
GSMUA_Achr8T11670_001	11.612357	#N/A	#N/A	NAD(P)H-quinone oxidoreductase subunit M
GSMUA_Achr7T11330_001	11.059776	#N/A	#N/A	Peroxidase 15
GSMUA_Achr4T31780_001	10.572519	#N/A	#N/A	Uncharacterized oxidoreductase
GSMUA_Achr3T17110_001	10.235707	#N/A	#N/A	Putative Estradiol 17-beta-dehydrogenase 12
GSMUA_Achr9T13520_001	10.167764	8.9080272	#N/A	Protein WAX2
GSMUA_Achr11T24060_001	10.089986	10.264349	#N/A	Tropinone reductase homolog
GSMUA_Achr11T01430_001	7.9841659	#N/A	#N/A	Ferredoxin--NADP reductase, leaf isozyme, chloroplastic
GSMUA_Achr11T22300_001	7.8215417	#N/A	#N/A	Glyceraldehyde-3-phosphate dehydrogenase B
GSMUA_Achr11T01900_001	7.0650247	#N/A	#N/A	Protochlorophyllide reductase B,
GSMUA_Achr7T07880_001	6.6074744	4.3293196	#N/A	Probable peroxisomal (S)-2-hydroxy-acid oxidase 2
GSMUA_Achr9T29540_001	6.3137197	#N/A	#N/A	Putative Peroxidase 12
GSMUA_Achr3T11750_001	6.2037078	9.1036267	10.2955	Putative 3-oxoacyl-[acyl-carrier-protein] reductase
GSMUA_Achr8T11740_001	6.1151529	7.4362729	6.9971128	Aldehyde dehydrogenase family 3 member F1
GSMUA_Achr6T00040_001	6.00509	#N/A	#N/A	Cytochrome P450 85A1
GSMUA_Achr5T20330_001	4.9459265	4.6176329	#N/A	Peroxidase 5
GSMUA_Achr8T12220_001	4.8864591	6.5639038	#N/A	fatty acid hydroxylase, putative,
GSMUA_Achr3T25050_001	4.6789856	#N/A	#N/A	Magnesium-protoporphyrin IX monomethyl ester [oxidative] cyclase, chloroplastic
GSMUA_Achr10T00370_001	4.2126663	#N/A	#N/A	Magnesium-protoporphyrin IX monomethyl ester [oxidative] cyclase
GSMUA_Achr4T14320_001	4.1942108	5.0695261	#N/A	UDP-glucose 6-dehydrogenase
GSMUA_Achr2T13890_001	3.6586487	#N/A	4.5179883	5'-adenylylsulfate reductase 3
GSMUA_Achr6T14620_001	3.5061891	5.4906648	#N/A	Putative Peroxidase 52
GSMUA_Achr10T12920_001	3.4141129	5.1445819	4.7591894	Trans-cinnamate 4-monooxygenase
**Cell wall related genes**				
GSMUA_Achr5T17150_001	#N/A	#N/A	7.501847	Hypothetical protein
GSMUA_Achr7T15620_001	#N/A	8.0811427	7.5249613	Pectinesterase/pectinesterase inhibitor PPE8B
GSMUA_Achr7T11480_001	#N/A	3.7920904	#N/A	Pectinesterase 3
GSMUA_Achr11T22960_001	#N/A	14.593784	17.486186	Expansin-A8
GSMUA_Achr5T07470_001	#N/A	10.714791	11.668475	Expansin-A2
GSMUA_Achr5T07480_001	#N/A	12.899815	12.492532	Expansin-A2
GSMUA_Achr6T10730_001	#N/A	6.1668	#N/A	Expansin-A15
GSMUA_Achr11T05430_001	#N/A	-5.706748	-5.044671	Pectinesterase 3
GSMUA_Achr1T23250_001	#N/A	#N/A	#N/A	Hevein-like protein
GSMUA_Achr6T21330_001	#N/A	#N/A	#N/A	Endochitinase PR4
GSMUA_Achr5T17150_001	#N/A	#N/A	7.501847	Hypothetical protein
GSMUA_Achr7T15620_001	#N/A	8.0811427	7.5249613	Pectinesterase/pectinesterase inhibitor PPE8B
GSMUA_Achr7T11480_001	#N/A	3.7920904	#N/A	Pectinesterase 3
GSMUA_Achr11T22960_001	#N/A	14.593784	17.486186	Expansin-A8
GSMUA_Achr5T07470_001	#N/A	10.714791	11.668475	Expansin-A2
GSMUA_Achr5T07480_001	#N/A	12.899815	12.492532	Expansin-A2
GSMUA_Achr6T10730_001	#N/A	6.1668	#N/A	Expansin-A15
GSMUA_Achr11T05430_001	#N/A	-5.706748	-5.044671	Pectinesterase 3
GSMUA_AchrUn_randomT04250_001	#N/A	8.9612034	9.1558624	Probable pectate lyase 15
GSMUA_Achr6T28260_001	#N/A	9.4929317	9.5099626	Probable pectate lyase 22
GSMUA_Achr4T26690_001	#N/A	7.0687309	#N/A	Probable pectate lyase 15
Heat shock protein				
GSMUA_Achr10T21120_001	-3.368716	-4.647048	#N/A	17.2 kDa class II heat shock protein
GSMUA_Achr4T11940_001	#N/A	3.3381916	#N/A	Luminal-binding protein 4
GSMUA_Achr8T23700_001	#N/A	4.4147024	#N/A	heat shock protein DnaJ, putative, expressed
GSMUA_AchrUn_randomT07470_001	#N/A	-3.700634	#N/A	heat shock protein DnaJ, putative, expressed
GSMUA_Achr7T05830_001	#N/A	7.5741686	9.6485957	Oligopeptide transporter 7
GSMUA_Achr3T31860_001	#N/A	#N/A	12.951674	heat shock transcription factor
aquaporin				
GSMUA_Achr11T00590_001	3.0154888	#N/A	#N/A	Aquaporin PIP2-3
GSMUA_Achr9T28940_001	#N/A	-4.190714	#N/A	Probable aquaporin TIP2-2
GSMUA_Achr4T20780_001	2.996134	3.9810613	#N/A	Aquaporin PIP2-4
GSMUA_Achr8T12920_001	#N/A	-4.358404	#N/A	Probable aquaporin TIP1-1
GSMUA_Achr6T05830_001	#N/A	-3.395407	#N/A	Probable aquaporin TIP2-2
GSMUA_Achr11T02240_001	3.8748548	#N/A	#N/A	Probable aquaporin TIP1-1
GSMUA_AchrUn_randomT06140_001	5.6947865	#N/A	5.65202	Probable aquaporin PIP1-2

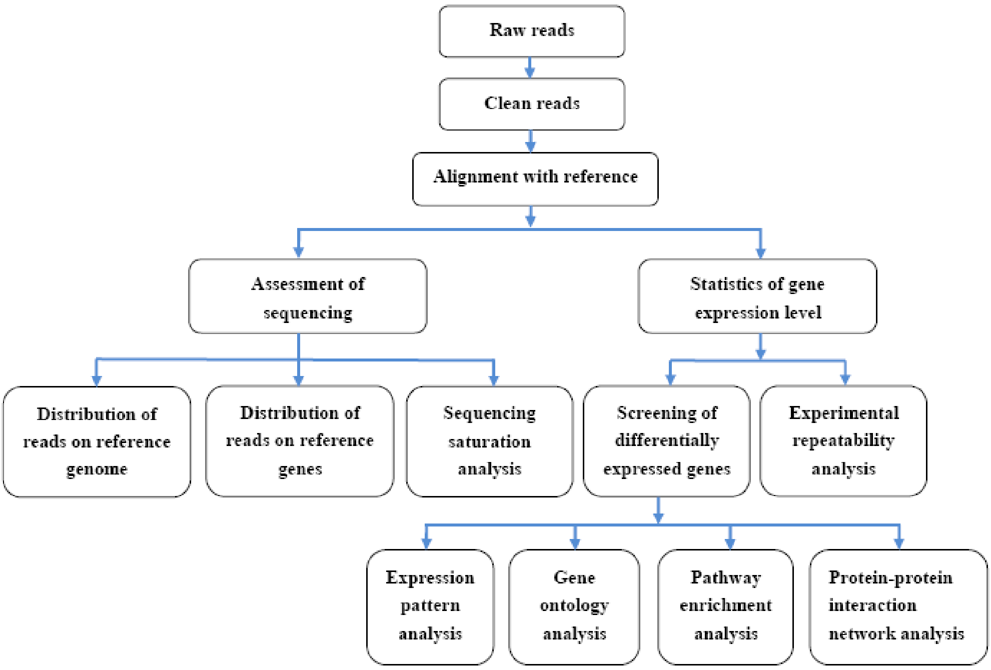

Figure S1. The pipeline of all the analysis done in this study.

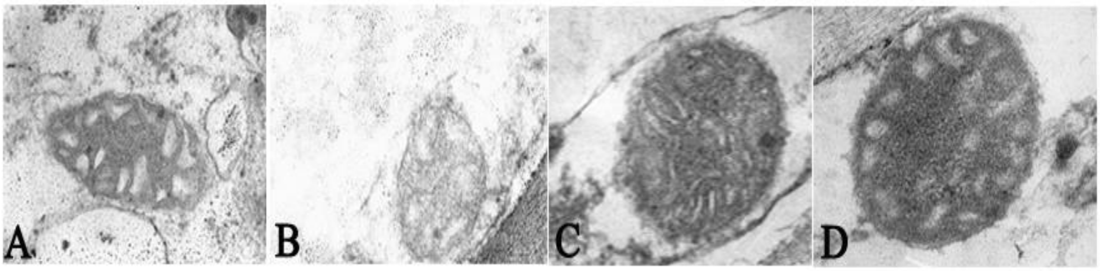

Figure S2. The ultrastructure of mitochondria of banana flesh cell in different ripening stages. **A**: F1; **B**: F2; **C**: F3; **D**: F4.

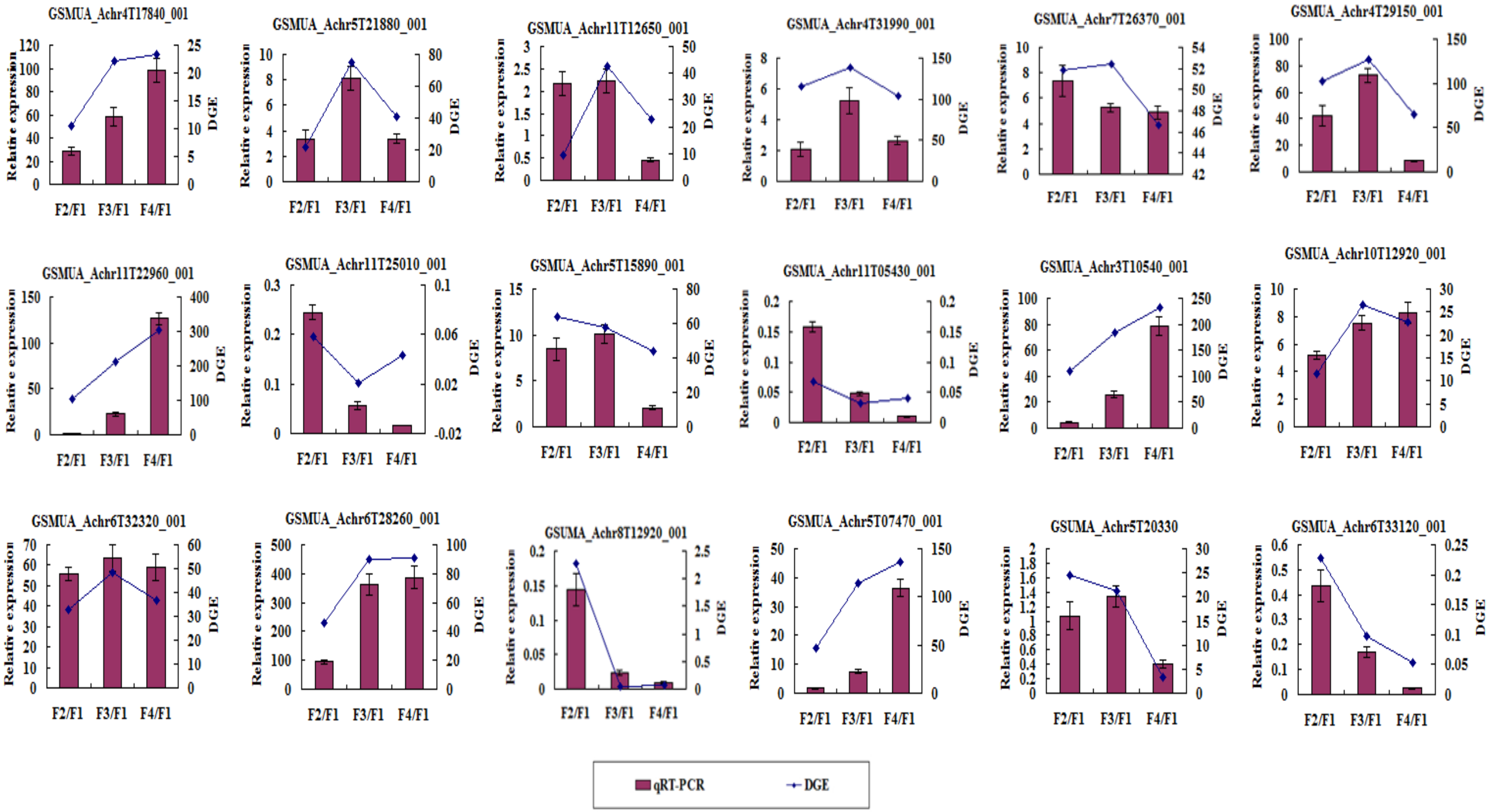

Figure S3. Validation of the RNA-Seq expression data using quantitative real-time PCR.

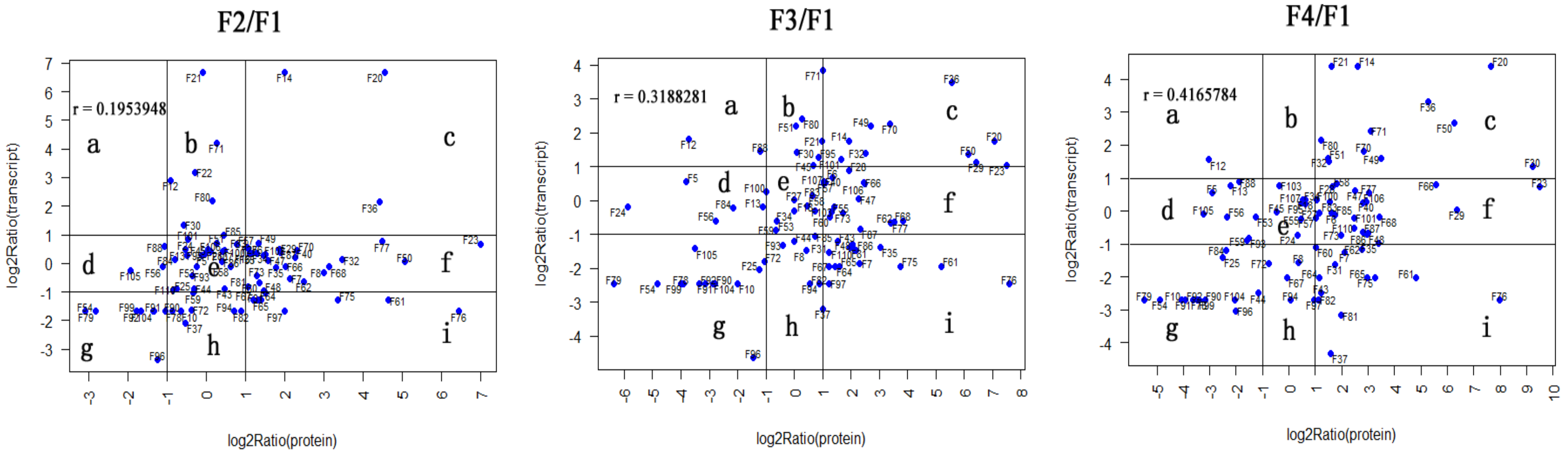

Figure S4. Correlation between the differentially expressed proteins and genes in the whole library. The x-axis shows the expression quantity of the differentially expressed proteins and the y-axis shows the expression quantity of genes in the whole library.

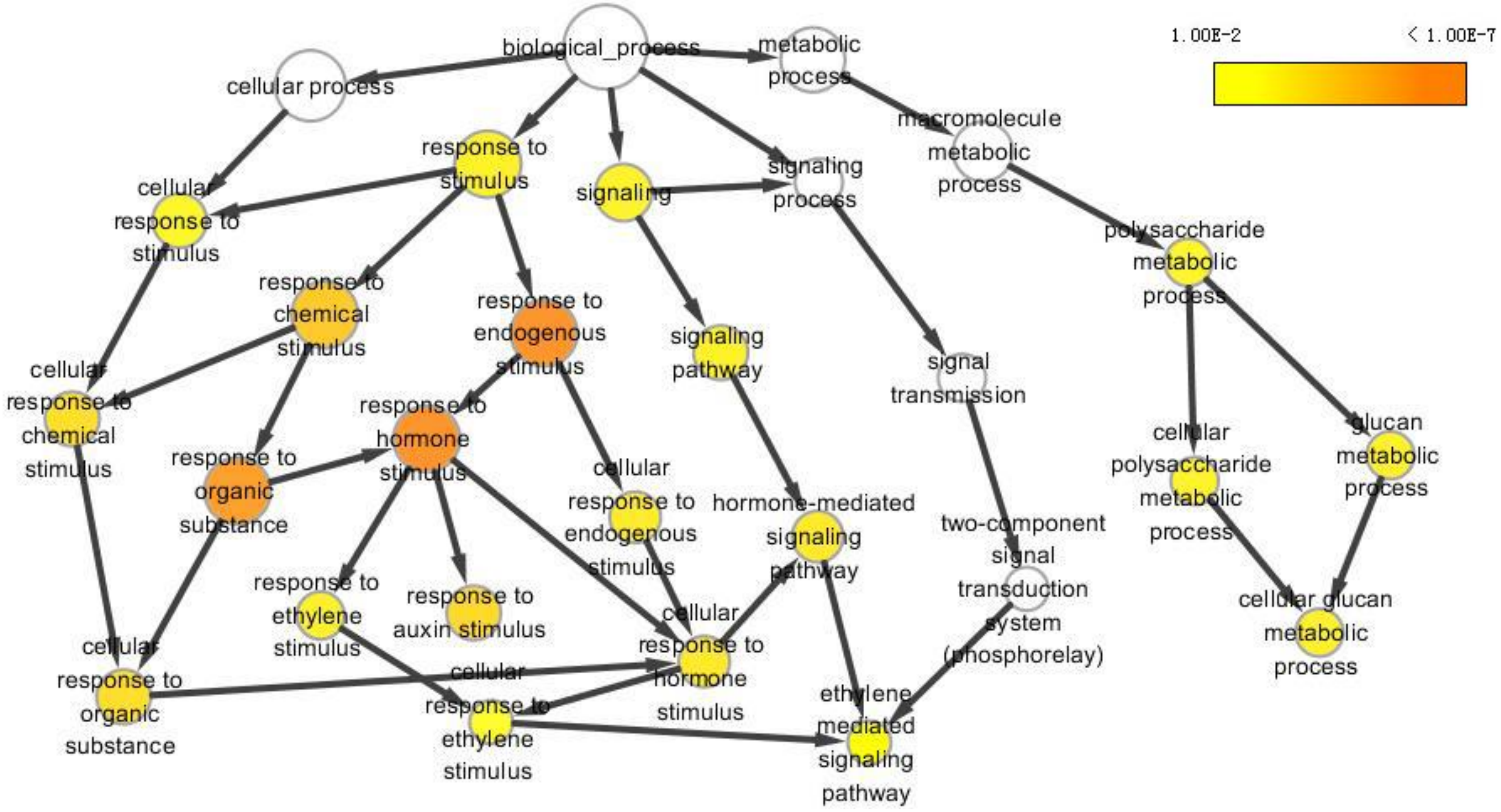

Figure S5. Clustering of signal transduction related genes using BiNGO 2.44.

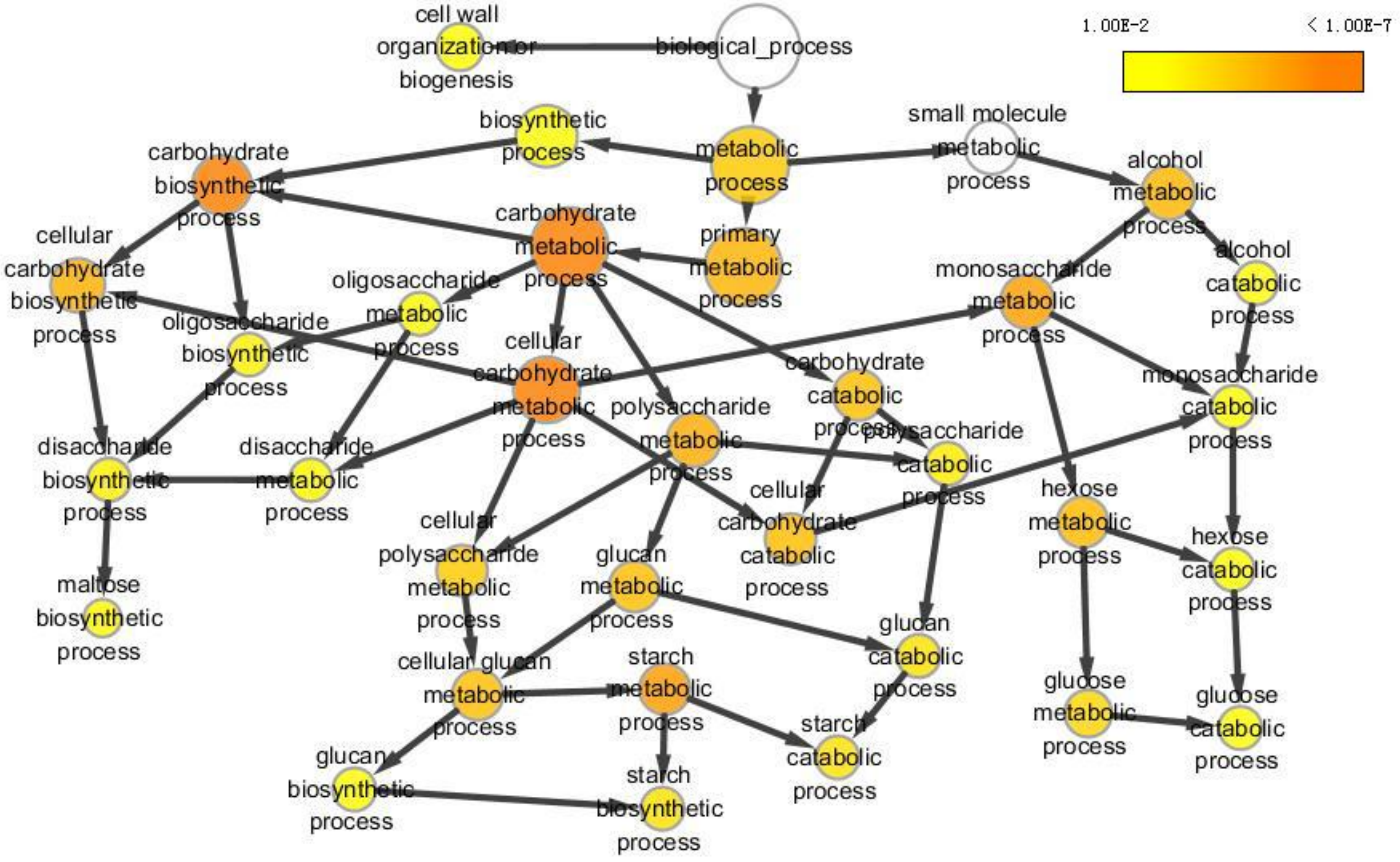

Figure S6. Clustering of sugar metabolism related genes using BiNGO 2.44.

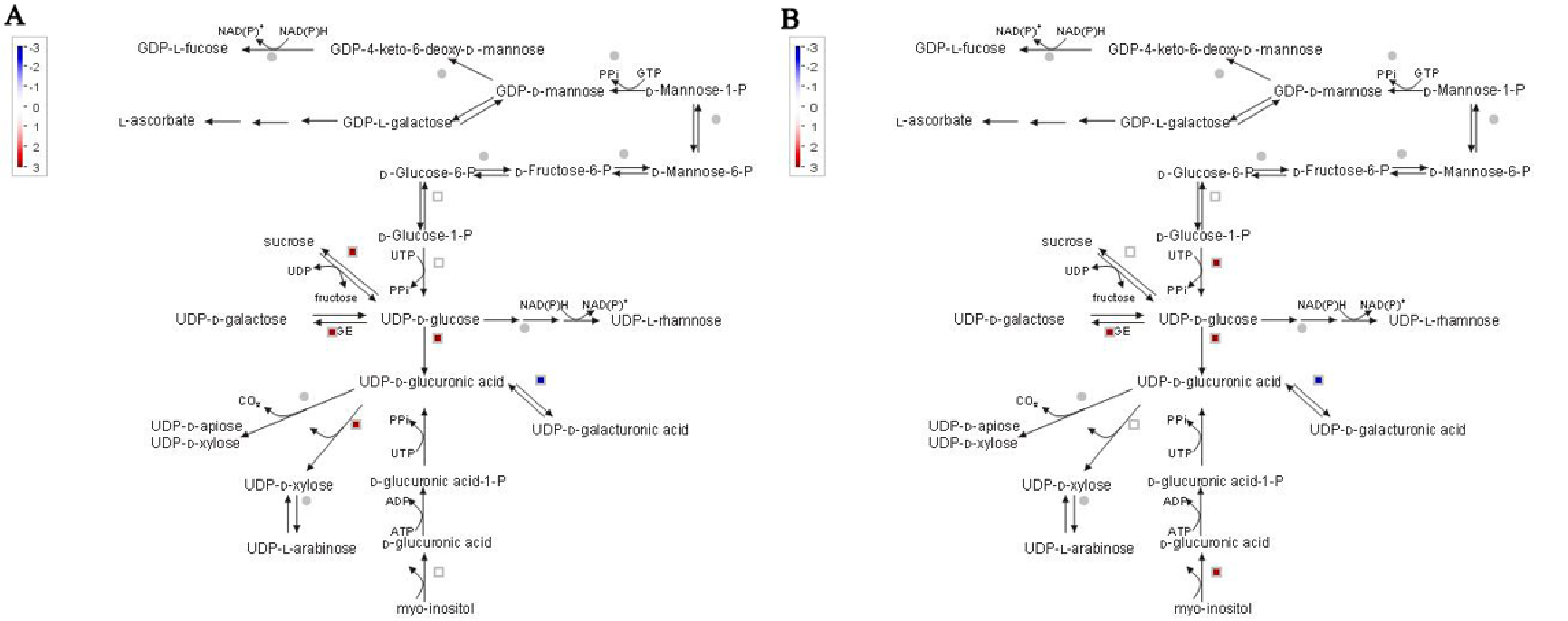

Figure S7. *Cont.*

C

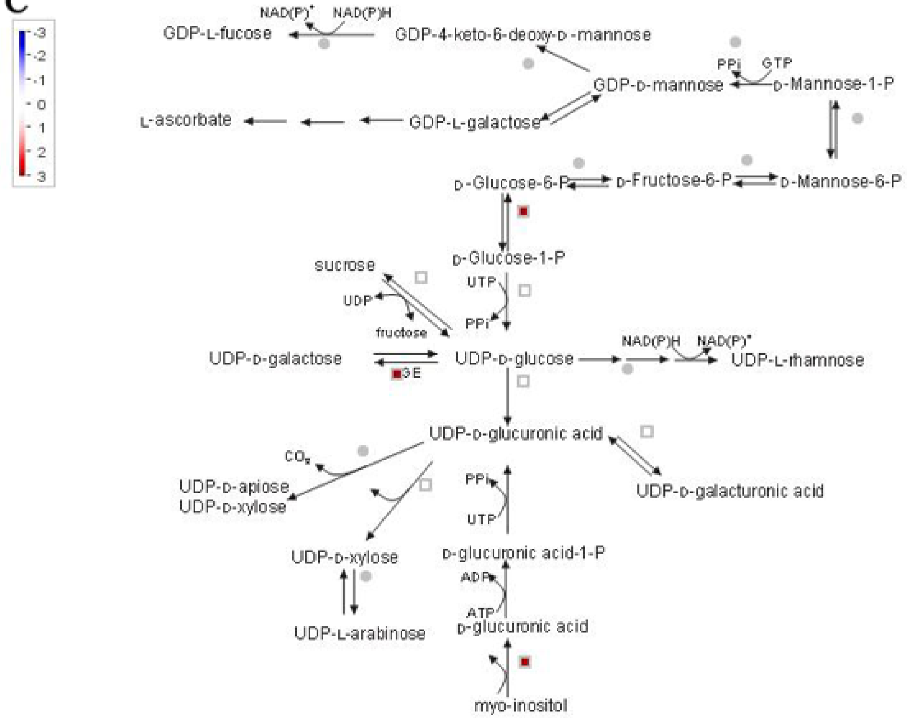

Figure S7. Schematic of the cell wall metabolism pathway using the MapMan visualization platform. The red or blue squares indicate the up- or down-regulated genes involved in the second metabolic pathways. **A**: F2/F1; **B**: F3/F1; **C**: F4/F1.

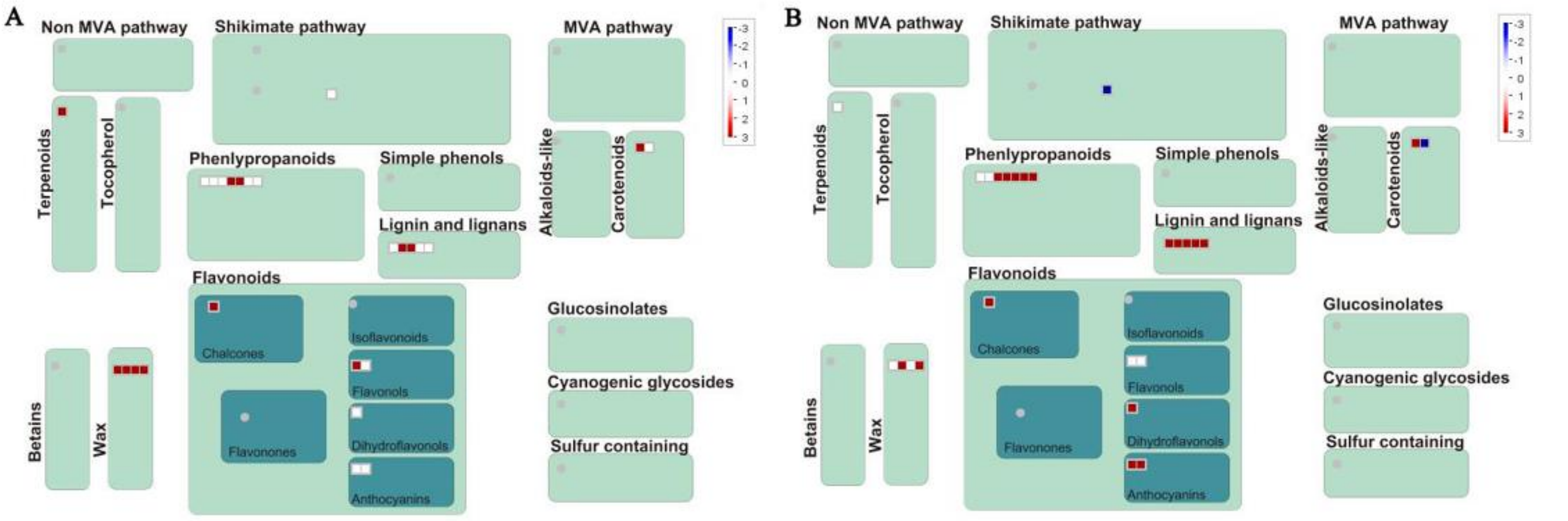

Figure S8. *Cont.*

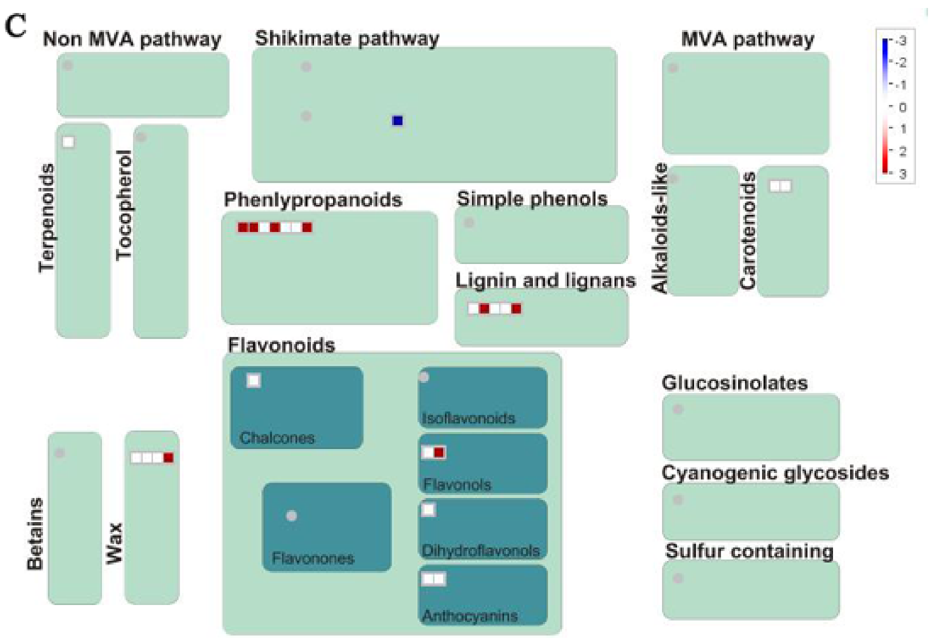

Figure S8. Schematic of the secondary metabolism pathway using the MapMan visualization platform. The red or blue squares indicate the up- or down-regulated genes involved in the second metabolic pathways. **A**: F2/F1; **B**: F3/F1; **C**: F4/F1.
